# Characterization of Yellow Fever Virus Infection of Human and Non-human Primate Antigen Presenting Cells and Their Interaction with CD4^+^ T Cells

**DOI:** 10.1371/journal.pntd.0004709

**Published:** 2016-05-18

**Authors:** Yu Cong, Monica A. McArthur, Melanie Cohen, Peter B. Jahrling, Krisztina B. Janosko, Nicole Josleyn, Kai Kang, Tengfei Zhang, Michael R. Holbrook

**Affiliations:** 1 National Institute of Allergy and Infectious Diseases (NIAID) Integrated Research Facility, Ft. Detrick, Frederick, Maryland, United States of America; 2 Department of Pediatrics and Center for Vaccine Development, University of Maryland, Baltimore, Maryland, United States of America; 3 Gears Inc., Lanham, Maryland, United States of America; University of Texas Medical Branch, UNITED STATES

## Abstract

Humans infected with yellow fever virus (YFV), a mosquito-borne flavivirus, can develop illness ranging from a mild febrile disease to hemorrhagic fever and death. The 17D vaccine strain of YFV was developed in the 1930s, has been used continuously since development and has proven very effective. Genetic differences between vaccine and wild-type viruses are few, yet viral or host mechanisms associated with protection or disease are not fully understood. Over the past 20 years, a number of cases of vaccine-associated disease have been identified following vaccination with 17D; these cases have been correlated with reduced immune status at the time of vaccination. Recently, several studies have evaluated T cell responses to vaccination in both humans and non-human primates, but none have evaluated the response to wild-type virus infection. In the studies described here, monocyte-derived macrophages (MDM) and dendritic cells (MoDC) from both humans and rhesus macaques were evaluated for their ability to support infection with either wild-type Asibi virus or the 17D vaccine strain and the host cytokine and chemokine response characterized. Human MoDC and MDM were also evaluated for their ability to stimulate CD4^+^ T cells. It was found that MoDC and MDM supported viral replication and that there were differential cytokine responses to infection with either wild-type or vaccine viruses. Additionally, MoDCs infected with live 17D virus were able to stimulate IFN-γ and IL-2 production in CD4^+^ T cells, while cells infected with Asibi virus were not. These data demonstrate that wild-type and vaccine YFV stimulate different responses in target antigen presenting cells and that wild-type YFV can inhibit MoDC activation of CD4^+^ T cells, a critical component in development of protective immunity. These data provide initial, but critical insight into regulatory capabilities of wild-type YFV in development of disease.

## Author Summary

Yellow fever virus (YFV) is a mosquito-borne flavivirus that can cause lethal hemorrhagic fever in infected humans. An effective live-attenuated vaccine, 17D, was developed in 1937 and continues to be used today. Over the past several years, a number of cases of vaccine-associated disease have been identified and linked to a compromised immune status. In the studies presented here we evaluated the susceptibility of macrophages and dendritic cells (DCs) to YFV infection, their cytokine response to infection and their ability to interact with and stimulate CD4^+^ T cells. These studies found that macrophages and DCs derived from both humans and non-human primates were susceptible to infection with either a wild-type Asibi YFV or the 17D vaccine virus, that the two viruses stimulated different cytokine responses in these cells and that human DCs infected with the 17D virus were able to stimulate CD4^+^ T cells while those infected with Asibi virus did not. These data demonstrate clear differences between the wild-type and vaccine viruses that may be related to the success of the 17D vaccine. These data also suggest that wild-type YFV may be able to inhibit critical components of the immune response to allow virus propagation and dissemination.

## Introduction

Yellow fever virus (YFV), the causative agent of yellow fever (YF), is a mosquito-borne flavivirus. YFV infection in humans can range from a sub-clinical infection to severe hemorrhagic fever and death. The currently available live-attenuated YFV vaccine, 17D, was developed in 1937 and over 500 million doses have been delivered. Vaccination with the 17D vaccine provides long-term protective immunity (at least 10 years) with a single immunization. Despite the existence of the 17D vaccine, YFV remains a significant human health concern. In fact, outbreaks of YF continue to occur in Africa and South America with an estimate of 130,000 cases per year in Africa alone with a case fatality rate of 60% in 2013[1].

While 17D is considered a safe and efficacious vaccine, over the past 20 years it has become increasingly apparent that occasional cases of vaccine-associated YF occur [2]. In cases of vaccine-associated disease from which virus was recovered, sequencing of the viral genome revealed it to be the vaccine strain 17D with no evidence of reversion to wild-type virus [3]. In some cases of vaccine-associated YF, there was evidence of underlying health issues potentially causing impaired immune status, particularly thymic disorders [3–5]. However, demonstration that co-morbidities contribute directly to development of disease is lacking. Given the apparent association of thymic disorders and impaired T cell immunity with vaccine-associated YF, the past several years have seen extensive efforts toward understanding the role of the T cell response following vaccination against YFV. These studies have largely focused on changes in T cell populations in vaccinated individuals and honing in on the criticality of the CD8^+^ T cell response following vaccination or infection of animals with the 17D vaccine strain [6–13]. Furthermore, evidence suggests limited B cell and CD8^+^ T cell responses may decrease vaccine efficiency in certain African populations [14]. Although fewer studies have focused on the CD4^+^ T cell response, CD4^+^ T cells play a critical role in development of a protective anti-YFV response and long-term immunological memory [15]. The few studies that are published have focused almost exclusively on the immune response to vaccination and not on the response to wild-type virus infection. Identification of differences between wild-type and vaccine virus infections is critical toward understanding mechanisms that viruses use to modulate the host immune response. In previous studies we found that wild-type YFV and live-attenuated 17D YFV elicited different responses in human hepatocytes and Kupffer cells. These studies demonstrated that infection with 17D YFV led to limited virus propagation and an immune response that would be indicative of rapid viral clearance and immune protection. On the other hand, the response to wild-type YFV infection was pronounced suggesting a “cytokine storm” that may be a critical component of the disease process [16, 17].

In the work presented here, we continued our investigation into the ability of YFV to regulate the host response to infection. Here, we asked whether carefully defined and highly purified subtypes of either human or rhesus macaque mature monocyte-derived dendritic cells (MoDC) or macrophages (MDM) were susceptible to infection with either the wild-type (Asibi) or vaccine (17D) strains of YFV. To determine if there were specific responses that differed between Asibi and 17D YFV infected cells, we measured cytokine secretion following *in vitro* viral infection. In addition, we evaluated the ability of infected human antigen presenting cells (APC) to stimulate CD4^+^ T cells in an effort to determine if YFV infection limited activation of cell-mediated immunity. We found that MoDC and MDM were susceptible to infection with either Asibi YFV or 17D YFV and that the kinetics of infection were consistent between human and non-human primate (NHP) derived cells. Interestingly, propagation of Asibi YFV was delayed in both MoDC and MDM relative to 17D YFV with kinetics suggesting decreased attachment efficiency. We also found that the cytokine response in human and NHP MoDCs was very limited, yet human DCs infected with 17D virus were effective at activating CD4^+^ T cells. Conversely, infection of human and NHP MDM stimulated a moderate cytokine response, but infected human MDM did not stimulate CD4^+^ T cells. These data demonstrate a central but differential role for DCs and macrophages as APC during Asibi virus infection and suggest that YFV inhibits intracellular response mechanisms that lead to activation of CD4^+^ T cells.

## Results

### Phenotype of APCs

Many studies evaluating the response and role of APCs in virus infection rely upon poorly defined or mixed populations of cells. A primary objective of our studies was to generate pure phenotypically homogeneous APCs. Confirmation of purity and viability was assessed by flow cytometric phenotyping. Our isolation and maturation protocols worked consistently for both human and NHP-derived cells with minimal inter-experimental variability.

**Fig 1:**
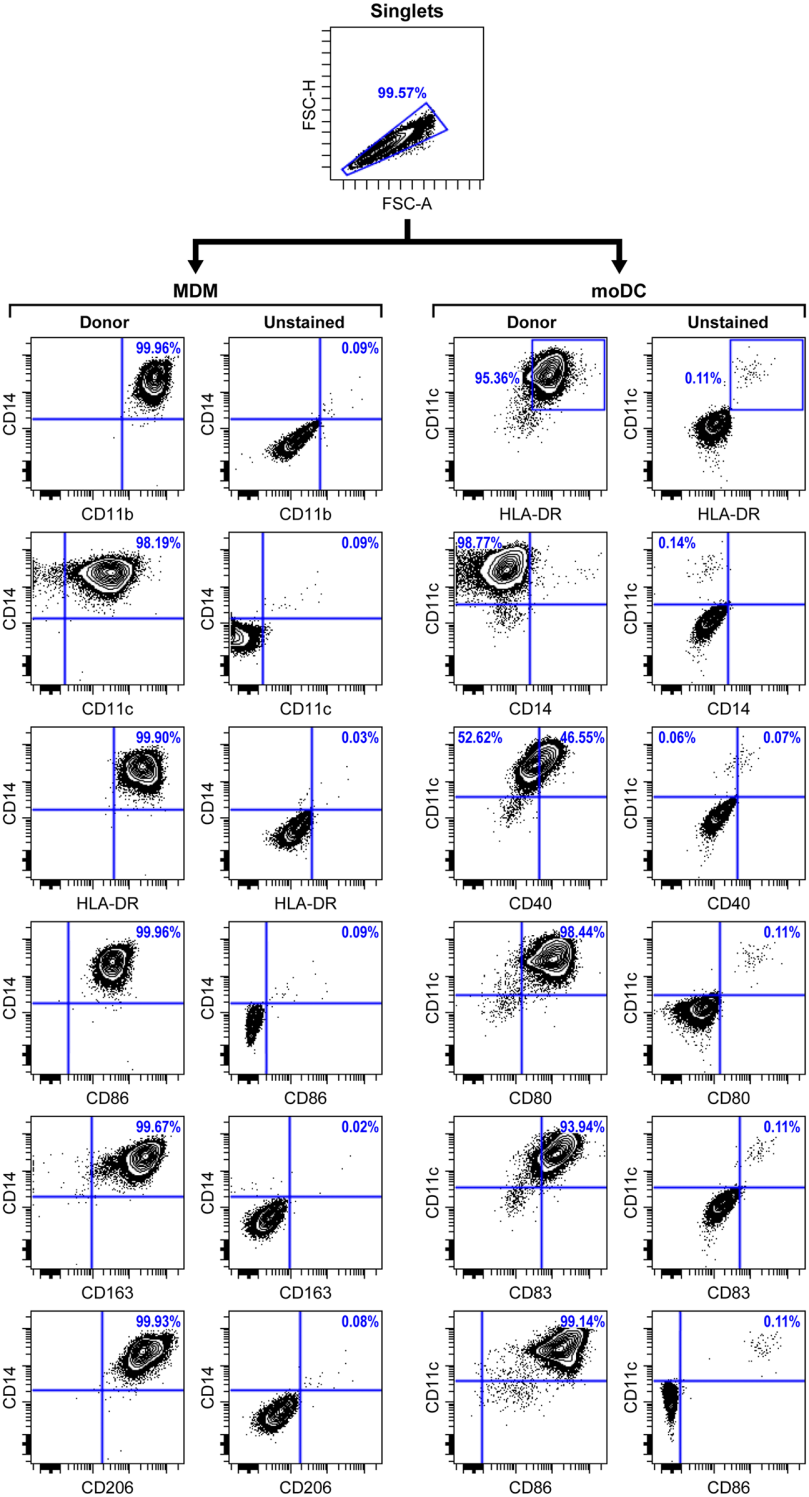
Gating strategy for phenotyping of MDM and MoDC. Following differentiation and maturation, MoDC and MDM were characterized by flow cytometry. The gating strategy included initial gating on monocyte populations from the FSC-A vs SSC-A plot, followed by exclusion of doublets and then gating on viable cells. Within the viable cell population, CD14 was plotted against all other markers for MDM gating and CD11c was plotted against all other markers for MoDC gating. An unstained control was used to set the gating for all plots. Shown here is an example of typical populations determined in the phenotyping analysis.

Characterization of human and NHP MDM showed that they were CD11b^+^, CD11c^+^, CD14^+^, CD86^+^, CD163^+^, CD206^+^, HLA-DR^+^, and CD83^-^ (Fig 1). Human and NHP MoDCs were CD11c^+^, CD80^+^, CD83^+^, CD86^+^, HLA-DR^+^ and CD14^-^ (Fig 1).

### Virus kinetics in APCs

Preliminary studies were completed to determine the susceptibility of immature NHP DC (imDC) and human bulk PBMC to infection with YFV 17D. Immature DCs supported replication in a manner similar to that seen in mature MoDCs while bulk PBMCs did not support YFV 17D infection and replication (S1 Fig). Due to challenges ensuring consistent generation of homogeneous populations of imDCs, continuing studies focused on mature cells.

**Fig 2:**
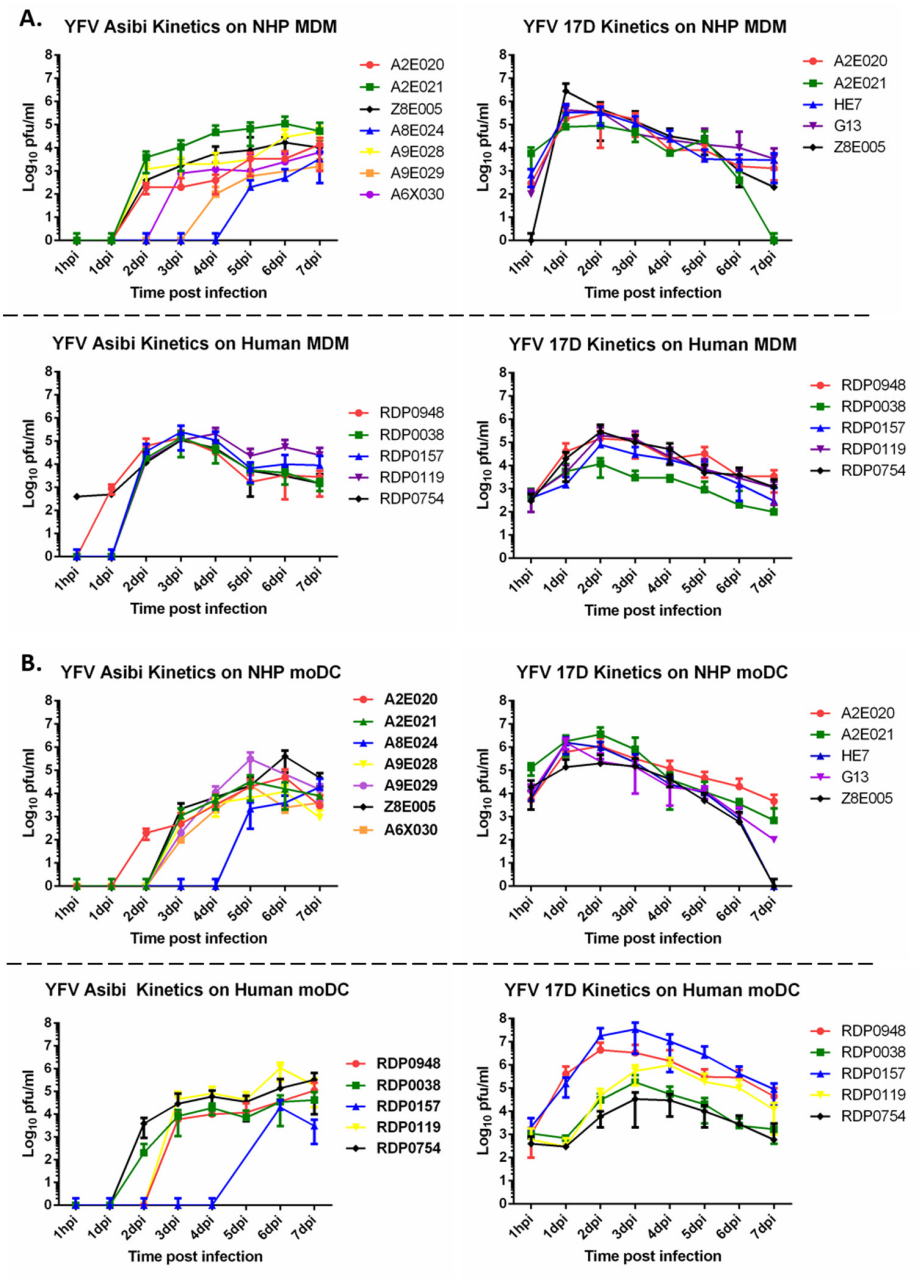
YFV kinetics in MDM and MoDC. Yellow fever propagation kinetics in NHP and human derived (A) MDM and (B) MoDC. Each line represents an individual donor and values in the legend provide the donor reference number. Titrations were performed in triplicate with data points representing the mean of the triplicate values.

In order to determine if YFV could replicate in MDM or MoDCs, cells were infected at a multiplicity of infection (MOI) of 0.1 and cell culture supernatants were collected immediately after virus adherence (i.e. 1 hour post-infection [hpi]) and daily until 7 days post infection (dpi). In MDM derived from both humans and NHP it was found that both wild-type Asibi virus and the vaccine strain 17D virus were able to infect and replicate within MDM based on evidence that viral titers in cells from each donor increased by at least 1 log_10_ subsequent to infection (Fig 2). The virus titers determined from supernatants collected at 1 hpi from 17D-infected MDM demonstrated titers in the 2–4 log_10_ range from all donors except from one NHP where the titer was less than 1 log_10_. In each set of donor MDM infected with 17D virus, peak titers ranged between 4–7 log_10_ 1–2 dpi. The kinetic profiles of 17D virus-infected MDM were virtually the same for all NHP donors and while the profiles were similar for human cells, titers in MDM from one human donor were about 2 log_10_ lower than MDM from other donors. Viral titers from NHP MDM infected with Asibi virus peaked at 3–5 log_10_ late in the infection cycle with significant donor-to-donor variation in the kinetics. The viral kinetics from human MDM following Asibi virus infection were virtually the same from all donors with a peak titer of around 5 log_10_ at 3–4 dpi. Interestingly, evidence of residual titer at 1 hpi seen in 17D virus infected MDM was not apparent in Asibi virus infected cells, possibly suggesting enhanced surface adherence of the 17D virus to MDM. In MDM tested from nearly all donors, both human and NHP, increases in viral titer following Asibi virus infection were not evident until at least 2 dpi, and in one instance as late as 5 dpi. These data suggest a restriction on wild-type Asibi virus replication in MDM, particularly those from rhesus macaques.

Kinetics studies with YFV-infected MoDCs produced results similar to those seen for YFV-infected MDM (Fig 2B). Both the 17D and Asibi viruses replicated in MoDCs with peak titers in 17D virus-infected NHP MoDCs in the range of 5–7 log_10_ and 4–8 log_10_ in human MoDCs. The kinetics in Asibi virus-infected MoDCs were delayed much like what was seen in Asibi virus-infected MDM, with increases in viral titer not typically seen until 2 dpi and as late as 5–6 dpi. Peak titers in both human and NHP MoDCs infected with Asibi virus ranged from 4–6 log_10_, regardless of the day of apparent replication onset.

As was seen in the MDM studies, there was a relatively high residual virus titer at 1 hpi in 17D virus-infected MoDC from both humans and NHP suggesting that the 17D virus may adhere more tightly to MDM and MoDCs than the Asibi virus. The delayed replication or poor adherence of the Asibi virus in MoDC, as was seen in MDM, also suggests a restriction in wild-type YFV internalization or replication that is not seen in 17D virus-infected cells.

To demonstrate that the residual virus was not an artifact, a small-scale study analyzing virus titers before and after a single wash step was performed on human MoDC and MDM from three donors. This study found that a single PBS wash could remove residual YFV Asibi from both MoDC and MDM while residual YFV 17D was around 3 log_10_ pfu/ml (S2 Fig). The efficiency of viral infection by YFV 17D and YFV Asibi could have a direct impact on subsequent biological processes, including cytokine responses and T cell interactions, as could residual or lightly adhered virus particles not removed by washing of cells.

In order to determine if structural differences between YFV 17D and Asibi could impact virus attachment and be responsible for the high residual titers in YFV 17D infected APC, we aligned amino acid sequence data from Beck et al [18] (Genbank Accession #s KF769016, KF769015) and submitted to the Swiss-Model modeling server (http://swissmodel.expasy.org/). We then compared the returned structures to previously published YFV NMR data from Volk et al [19] These models demonstrated five amino acid residue differences at the surface of the viral receptor-binding domain (Envelope protein, domain III). These changes are significant as they change the residue size, charge or polarity (e.g. Phe305Ser or Ser325Pro) and could directly impact the interaction between the virus and its receptor on the surface of APC (S3 Fig).

### Cytokine and chemokine production by YFV-infected APCs

In order to evaluate the reactivity of APCs to YFV infection and to determine if specific differences existed between the response to Asibi and 17D virus infections, we measured the release of a panel of cytokines and chemokines from virus- or mock-infected cells. In addition, we made specific comparisons between APCs derived from humans and those derived from NHP to identify similarities or differences between the human condition and the best animal model for YFV infection. Many of the cytokines or chemokines that were evaluated demonstrated a negligible change relative to mock-infected cells.

#### Human macrophages

**Fig 3:**
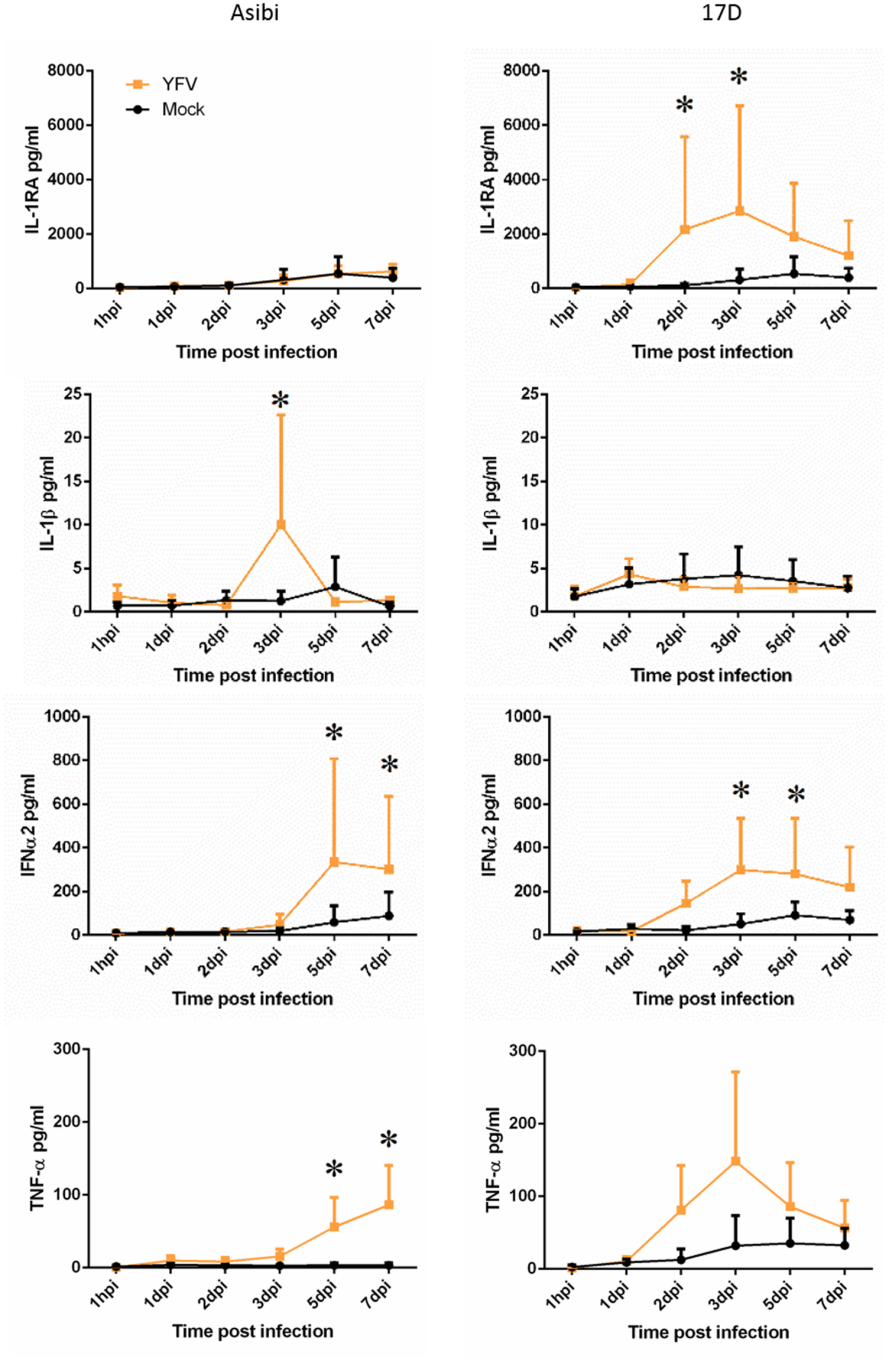
Cytokine response in human MDM. Cytokine response in human MDM infected with either wild-type Asibi virus or the vaccine strain 17D virus (yellow) in relation to mock (black) infected cells. All data points represent triplicate biological replicates. (*) indicates points of significant (p<0.05) difference between virus and mock infected cells. Repeated measures ANOVA run in SAS was used to determine statistical significance.

**Fig 4:**
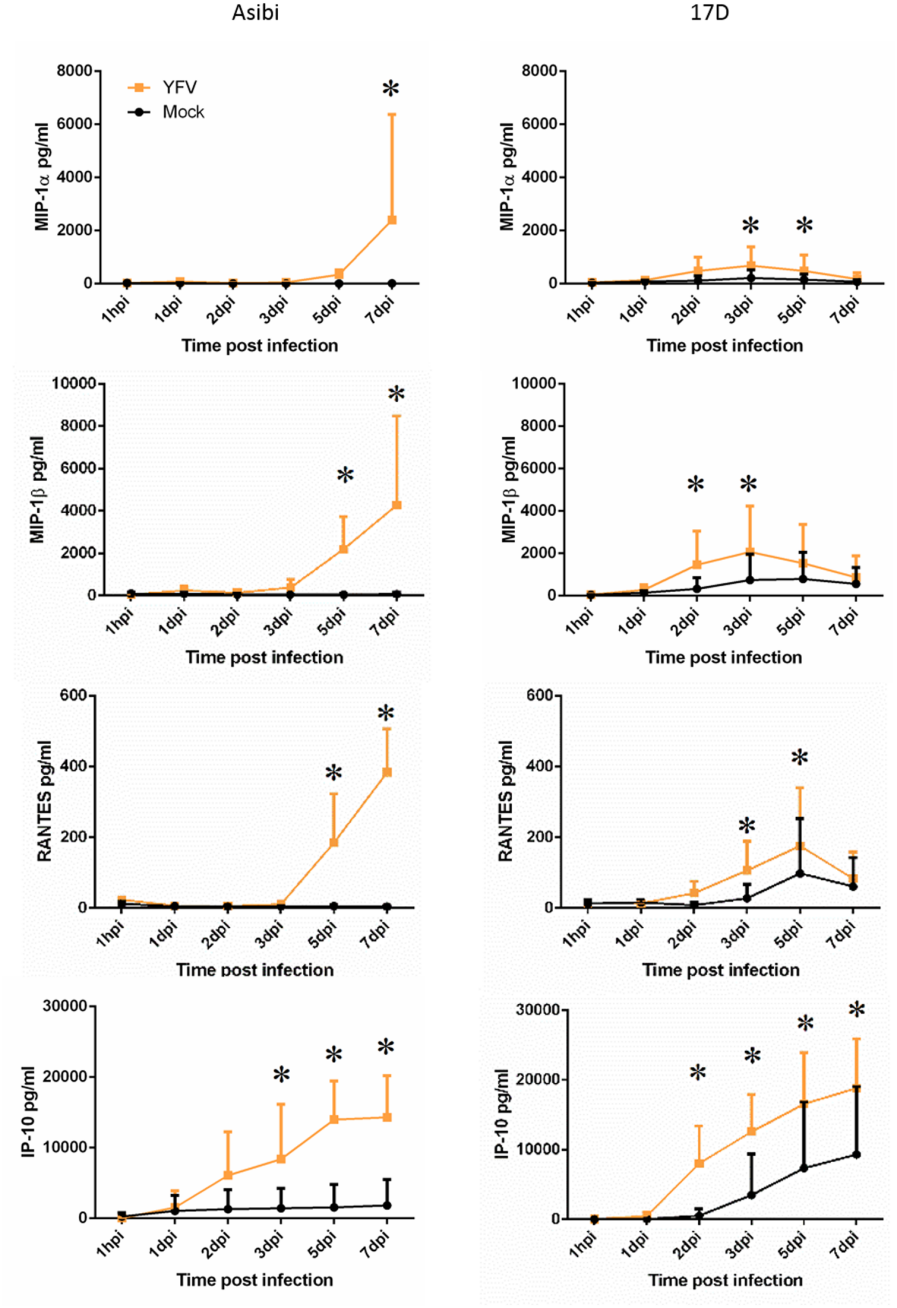
Chemokine response in human MDM. Chemokine response in human MDM infected with either wild-type Asibi virus or the vaccine strain 17D virus (yellow) in relation to mock (black) infected cells. All data points represent triplicate biological replicates. (*) indicates points of significant (p<0.05) difference between virus and mock infected cells. Repeated measures ANOVA run in SAS was used to determine statistical significance.

Evaluation of the cytokine response to 17D virus infection in human MDM found evidence of a response that peaked around 3 dpi, but that diminished over the course of the infection. In Asibi virus-infected cells, the cytokine response was delayed and generally did not diminish as the infection proceeded. In human MDM, IFNα2 and TNFα were both elevated 2–5 dpi in 17D-infected cells, but the response diminished as the infection proceeded (Fig 3). This is in contrast to Asibi virus-infected cells where the response persisted and increased as the infection progressed. IL-1RA was also elevated 2–5 dpi in 17D virus-infected MDM while there was no elevation in Asibi virus-infected MDM, suggesting a controlled anti-inflammatory response. The chemokine response evaluated through MIP1α, MIP1β and RANTES expression also suggested a controlled response to 17D virus infection with elevated expression 2–5 dpi while expression in Asibi virus-infected MDM was delayed and significantly elevated late in the infection (Fig 4). Expression profiles of IP-10 were similar in MDM infected with either Asibi or 17D viruses (Figs 3 and 4).

#### Human dendritic cells

**Fig 5:**
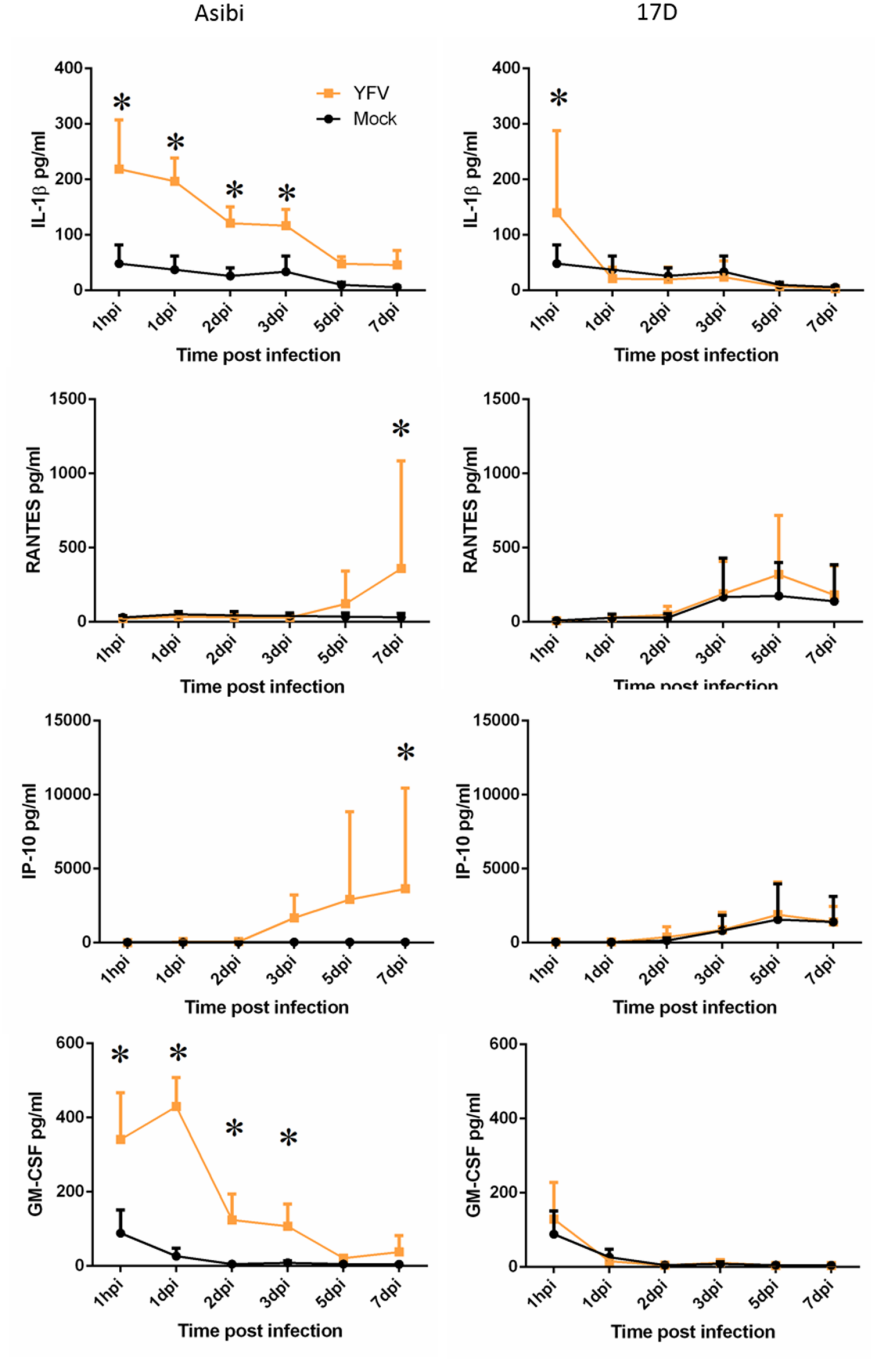
Cytokine and chemokine response in human MoDC. Cytokine and chemokine response in human MoDC infected with either wild-type Asibi virus or the vaccine strain 17D virus (yellow) in relation to mock (black) infected cells. (*) indicates points of significant (p<0.05) difference between virus and mock infected cells. Repeated measures ANOVA run in SAS was used to determine statistical significance.

Human MoDC had a largely muted response to YFV infection with the levels of most cytokines not changing significantly relative to mock-infected cells (Fig 5). Levels of IL-1β and GM-CSF were both elevated significantly from 1 hpi with wild-type Asibi virus until 3 dpi. The response in 17D virus-infected cells was not apparent except for elevated levels of IL-1β at 1 hpi (Fig 5) suggesting an attachment-mediated release of IL-1β that promptly decreased in 17D virus-infected cells, but that persisted with decreasing levels in Asibi virus infection. The chemokine response in YFV-infected human MoDC was also limited as only IP-10 and RANTES were elevated in Asibi virus-infected MoDC at dpi 5–7 with the response at 7 dpi statistically significant. There were no significant differences in the chemokine response between mock- and 17D virus-infected cells (Fig 5).

#### NHP macrophages

**Fig 6:**
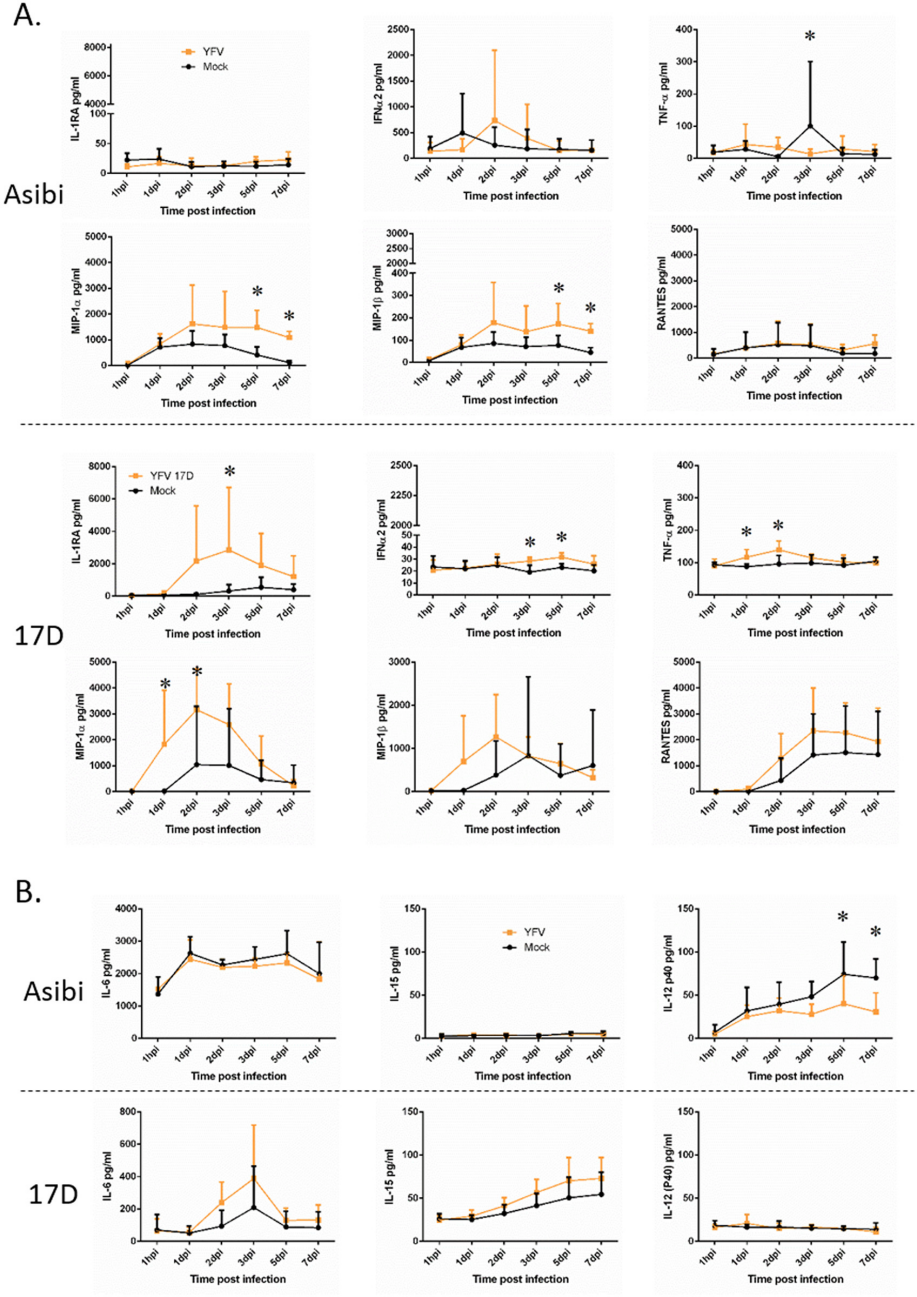
Cytokine and chemokine response in NHP MDM and MoDC. Cytokine and chemokine response in NHP (A) MDM or (B) MoDC infected with either wild-type Asibi virus or the vaccine strain 17D virus (yellow) in relation to mock (black) infected cells. (*) indicates points of significant (p<0.05) difference between virus and mock infected cells. Repeated measures ANOVA run in SAS was used to determine statistical significance.

The response to infection of NHP MDM was similar to that seen in human MDM where there were elevated levels of several cytokines in response to 17D virus infection, but a negligible or limited response to Asibi virus infection. Specifically, IL-1RA, IFNα2 and TNFα were all elevated within the first five days of infection relative to mock-infected controls, but there was little difference in expression between Asibi virus- and mock-infected MDM (Fig 6A). Chemokines MIP1α and MIP1β were both elevated early following infection with 17D virus, but then returned to levels comparable to mock-infected cells as the infection progressed. As in human MDM, infection with Asibi virus stimulated production of MIP1α and MIP1β later in the infection with statistically significant increases relative to mock-infected cells. Levels of RANTES, however, were elevated dpi 2–7 in 17D virus-infected cells and on dpi 7 in Asibi virus-infected cells, but these differences were not statistically significant relative to mock-infected cells (Fig 6A). As was the case in human MDM, these data suggest differential regulation of cytokine and chemokine release by the 17D and Asibi viruses.

#### NHP dendritic cells

The cytokine response in YFV-infected NHP MoDC was minimal and there were no changes in chemokine release levels relative to mock-infected cells. Both IL-6 and IL-15 were elevated in 17D virus-infected MoDC relative to mock-infected cells, but these data were not statistically significant. There were no changes in either IL-6 or IL-15 levels in Asibi virus-infected cells (Fig 6B). Interestingly, in Asibi virus-infected MoDC there was apparent inhibition of IL-12(p40) relative to mock-infected cells. IL-12(p40) is constitutively released from mock-infected MoDC, but this release appeared to be inhibited at dpi 5 and 7 following Asibi virus infection (Fig 6).

### T cell re-stimulation assays

**Fig 7:**
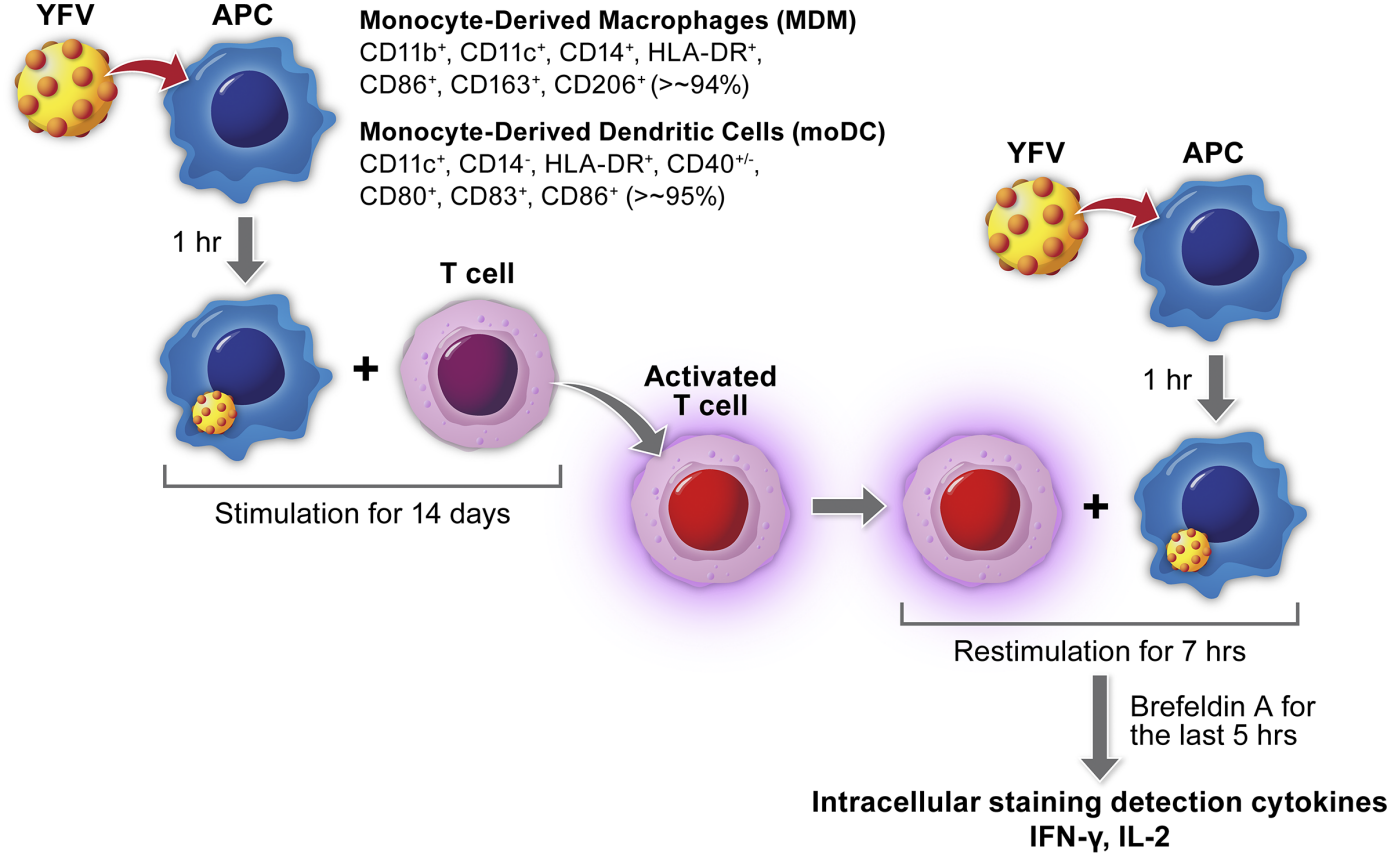
Experimental design of T cell re-stimulation studies. Design and work-flow of re-stimulation assays. YFV treated/infected APC are co-cultured with naïve autologous CD4^+^ T cells for 14 days prior to re-isolation of the now stimulated T cells. The stimulated T cells are then co-cultured with newly treated/infected APC for 7 hours before the T cells are isolated and intracellular cytokine production (IFN-γ and IL-2) is measured by flow cytometry.

**Fig 8:**
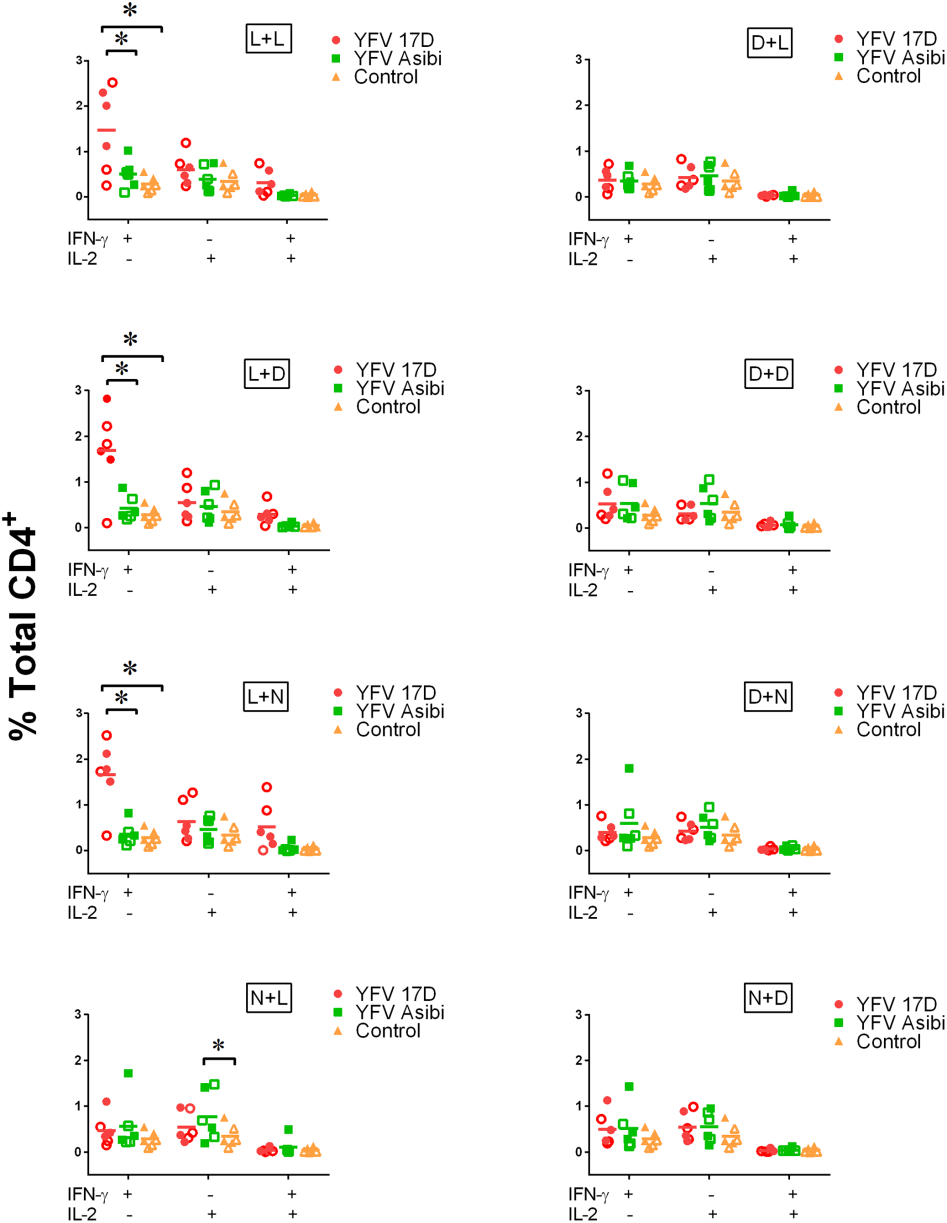
Cytokine response in CD4^+^ T cells: Wild-type Asibi virus vs. vaccine 17D virus infection. IFN-γ and IL-2 production by human CD4^+^ T cells in re-stimulation assays. Each data point represents the response from an individual donor (n = 6) with the horizontal bar indicating the mean of the six values. Red data points indicate 17D YFV-treated cells, green squares indicate Asibi YFV-treated cells and yellow triangles indicate mock-treated cells. Closed data points indicate cells from unvaccinated donors while open data points indicate cells from vaccinated donors. (L) indicates treatment with live virus, (D) indicates treatment with gamma-irradiated inactivated virus and (N) indicates mock-treated MoDC prior to co-culturing with CD4^+^ T cells (See Fig 7 and Materials and Methods). (*) indicates points of significant (p<0.05) difference between the indicated datasets (bracket). A non-parametric multi-T test was used to determine statistical significance.

Re-stimulation studies were carried out only with human samples due to a limited availability of NHP material. To evaluate the ability of infected MoDC to stimulate CD4^+^ T cells we infected MoDC with YFV and co-cultured them with autologous CD4^+^ T cells at a 1:60 ratio of MoDC:T cells. After two weeks of expansion, CD4^+^ T cells were re-stimulated with freshly YFV-infected MoDC. As a measurement of CD4^+^ T cell activation following stimulation, intracellular production of IFN-γ and IL-2 was measured by flow cytometry. In order to determine if active MoDC infection was required for CD4^+^ T cell stimulation, T cells were stimulated once or twice with either live (L) or inactivated (gamma irradiated)(dead) (D) YFV (Fig 7). CD4^+^ T cells initially co-cultured with MoDC + live YFV 17D demonstrated a significant increase in the percentage of IFN-γ^+^ T cells compared to unstimulated controls regardless of the re-stimulation conditions used (Fig 8). However, no significant increase was observed in percentages of either IFN-γ^+^ IL-2^+^ double positive or IL-2^+^ T cells. In contrast, CD4^+^ T cells initially co-cultured with MoDC + no virus followed by re-stimulation with MoDC + live Asibi (7 hours) resulted in a significant increase in the percentage of IL-2-producing T cells compared to unstimulated controls (Fig 8). T cells stimulated with MoDC + inactivated YFV (17D or Asibi) did not show significant increases in the percentages of cytokine-producing T cells compared to unstimulated controls.

**Fig 9:**
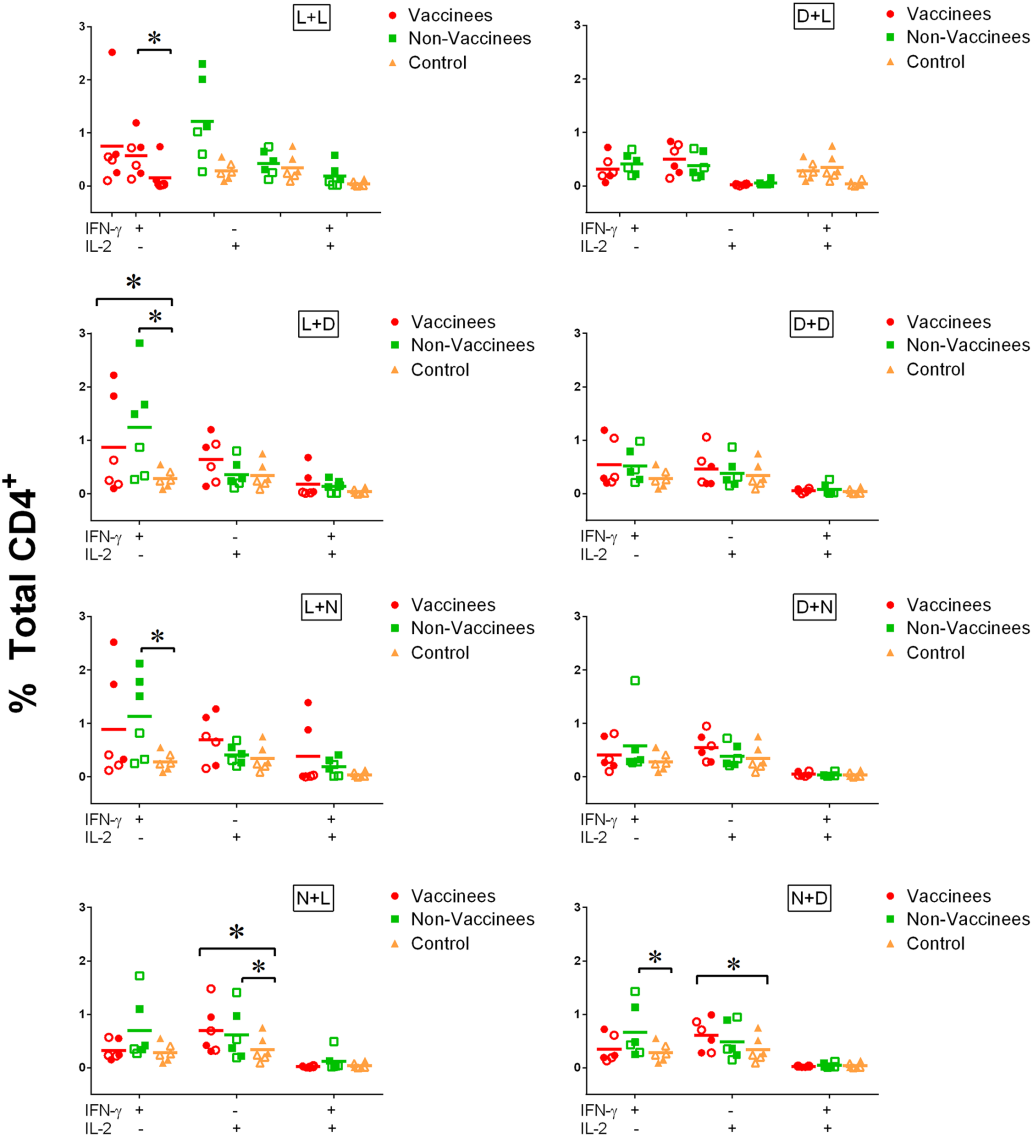
Cytokine response in CD4^+^ T cells: Vaccinated vs. unvaccinated. IFN-γ and IL-2 production by human CD4^+^ T cells in re-stimulation assays. Each data point represents the response from an individual donor (n = 6) with the horizontal bar indicating the mean of the six values. Red circles indicate cells isolated from vaccinated donors and green squares indicate cells isolated from unvaccinated donors. Yellow triangles indicate mock-treated (N+N) control cells. Closed data points indicate cells infected with YFV 17D while open data points indicate cells infected with YFV Asibi. (L) indicates treatment with live virus, (D) indicates treatment with gamma-irradiated inactivated virus and (N) indicates mock-treated MoDC prior to co-culturing with CD4^+^ T cells (See Fig 7 and Materials and Methods). (*) indicates points of significant (p<0.05) difference between the indicated datasets (bracket). A non-parametric multi-T test was used to determine statistical significance.

Half of the donors tested in these studies were previously vaccinated with the 17D vaccine. In order to determine if vaccination impacted IFN-γ or IL-2 production by CD4^+^ T cells, data were analyzed based on vaccination status (Fig 9). As with the analyses based on virus strain, stimulation with MoDC + inactivated virus had no significant impact on IFN-γ or IL-2 production, while infection with MoDC + live virus resulted in increased percentages of cytokine-producing T cells. Although significant differences relative to unstimulated controls were observed, there were no significant differences in either IFN-γ or IL-2 production between vaccinated and unvaccinated donors.

Control, unstimulated, CD4^+^ T cells retained >95% viability over the course of the study (Table 1). T cells stimulated with Asibi virus also maintained >95% viability while viability of CD4^+^ T cells stimulated with the 17D virus was considerably lower with average viabilities of 72–74%. However, T cells initially stimulated with MoDC + inactivated 17D virus had viability >93% which is similar to cells stimulated only once with either live or inactivated virus (i.e. N+L or N+D) (Table 1). These data suggest that stimulation with MoDC infected with replicating YFV 17D has a negative impact on the viability of CD4^+^ T cells in this co-culture system while stimulation with MoDC infected with wild-type Asibi virus had negligible impact on CD4^+^ T cell viability. Interestingly, while viability was somewhat lower, CD4^+^ T cells co-cultured with MoDC + live 17D demonstrated the most pronounced increase in the percentage of cytokine-producing cells compared to unstimulated controls. There were no consistent differences in viability among donors naïve for YFV and those who had previously been vaccinated.

**Table 1:** CD4^+^ T cell viability following co-culture with MoDC.

	Human CD4+ T cell Percent (%) Viability																
	Control	YFV 17D								YFV Asibi							
Donor	N+N	L+L	L+D	L+N	D+L	D+D	D+N	N+L	N+D	L+L	L+D	L+N	D+L	D+D	D+N	N+L	N+D
RDP0038	80.5		99.8	99.7	99.8	99.8	99.8	80.3	80.6	99.8	99.8	99.7	99.8	99.8	99.6	81.3	79.7
RDP0754	94.9	92.0	89.1	87.1	73.9	71.6	83.4	54.3	58.1	77.2	80.7	61.8	79.0	91.0	90.7	94.9	54.3
RDP0890	85.1	75.7	78.2	76.2	72.8	71.1	71.7	73.8	76.0	77.1	72.7	79.2	83.3	82.7	81.3	78.4	75.7
RDP0948	99.7	99.8	99.8	99.8	99.8	99.8	99.7	87.7	88.0	99.7	99.7	99.8	99.7	99.6	99.7	87.2	86.6
RDP0583	41.0	16.6	35.6	45.1	51.4	43.5	45.7	79.4	82.2	36.2	27.8	38.7	40.9	36.1	39.1	75.6	78.0
RDP0157	99.7	99.8	99.8	99.7	99.7	99.8	99.8	72.7	71.1	99.6	99.7	99.7	99.7	99.7	99.7	70.8	71.9
Avg	83.5	76.8	83.7	84.6	82.9	80.9	83.4	74.7	76.0	81.6	80.1	79.8	83.7	84.8	85.0	81.4	74.4

Evaluation of CD4^+^ T cell viability in co-culture with MoDC. N = No antigen stimulation; L = Live virus stimulation; D = Dead (irradiated) virus stimulation using either vaccine strain 17D or wild-type Asibi viruses in six individual human donors.

Similar studies were carried out using MDM stimulation of CD4^+^ T cells. In these studies there was no evidence of CD4^+^ T cell stimulation by APCs treated with either 17D or Asibi viruses (S4 and S5 Figs).

## Discussion

The importance of adaptive immunity following vaccination is well recognized, but in the case of flavivirus infections, the focus on adaptive immunity has largely been toward the development of an antibody response. Historical definitions of protective immunity against flaviviruses have identified a 1:10 neutralizing antibody titer as protective. Over the past few years a greater appreciation of the importance of the cell-mediated response in flavivirus infections has become evident. However, for YFV infection, comparatively little is known about the role of APC and T cells in protection against, or development of, disease. Recently, a number of groups have been evaluating a range of host response parameters in human YFV vaccine trials and found that the human T cell response is significant and appears critical toward development of protective immunity following vaccination. As might be expected, vaccination with the 17D vaccine induced effector CD4^+^ T cell responses with a peak around 2 weeks post-vaccination then transitioning to a central memory response [15]. Further studies have shown that CD8^+^ T cells also play a role in the response to vaccination, whether by clearing the vaccine virus or in development of a sustained memory response detectable up to 25 years post-vaccination [10, 12, 20]. Clearly the role of T cells in development of a protective response following vaccination is critical, but none of these studies address the potential role of T cells, or inhibition of a T cell response, in an acute wild-type virus infection.

The role of APCs in the stimulation of an effective T cell response is critical for the development of protective immunity. Previous work has shown that YFV 17D can replicate in human DCs, does not induce maturation of immature DCs and can stimulate CD4^+^ and CD8^+^ T cells [21, 22]. In the studies described here, we strove to expand upon previous efforts with direct comparisons between infection of APCs with the 17D virus or wild-type YFV. Our objective was to identify specific differences in the response to infection indicative of direct viral regulation of the development of protective immunity. Initial studies were also performed to identify parallels between the response of NHP-derived APC and those derived from humans in an effort to enhance our understanding of the rhesus macaque as a model for YFV infection in humans.

Replication kinetics in MDM and MoDC from both human and NHP found that these cells were susceptible to infection with both wild-type Asibi YFV and the vaccine strain 17D. In human MDM and MoDC there was little variability between donors when infected with the 17D virus, but there was considerable variability when infected with the wild-type Asibi virus. Interestingly, in either MoDC or MDM from both humans and NHP, the kinetics profiles were consistent in cells infected with the 17D virus with increased titers at 1 dpi and peak titers 2–3 dpi. In cells infected with Asibi virus, there was frequently a considerable delay in virus replication, on some occasions up to 4–5 dpi before virus titers could be measured. In these studies, the data presented are compilations of several individual experiments performed independently. A single study with three donors also clearly demonstrated that YFV Asibi could easily be washed from the MoDC and MDM. These data suggest a restriction on wild-type YFV replication or efficiency of infection that is not present in 17D virus infected cells. This restriction may be critical to limiting an innate response to virus infection and to provide for efficient virus dissemination. In addition, the restriction identified in these experiments could have a direct impact on the results of these studies as the specific number of virus particles entering the target cells during the 1 hour infection cannot be quantified, but is likely different between YFV 17D and YFV Asibi. Previous studies have shown that potential attachment proteins, such as DC-SIGN and some integrins, do not facilitate virus entry into DCs [21]. The data provided here suggests that differences in virus attachment and entry may be associated with structural or chemical differences between the wild-type and 17D viruses. Glycosylation of the viral envelope protein should be consistent between virus stocks as both were generated in Vero cells. The 17D vaccine strain differs from its parental wild-type strain Asibi by only 32 amino acids in a 3411 amino acid open reading frame with seven occurring in the viral envelope protein, several of which are in the putative receptor-binding domain [18, 23]. Structural analysis of the YFV E protein domain III demonstrated significant amino acid differences between YFV 17D and Asibi changes that could impact virus attachment and entry. These differences in the amino acid sequence of the receptor-binding domain could potentially impact interactions with receptors on MDM or MoDCs. Recently, Fernandez-Garcia et al have shown that the differential attachment and internalization between YFV 17D and Asibi is due to differences in the endocytic pathway used by the viruses to infect host cells [24]. These studies, which focused on immortalized cell lines and immature MoDC, found that YFV Asibi utilizes a clathrin-mediated endocytic pathway while YFV 17D utilizes a clathrin-independent pathway. Fernandez-Garcia et al suggested that differences in the virus structural proteins could impact the choice entry pathway. Our modeling data suggests that receptor attachment may be the critical factor for determining the virus entry mechanism.

Evaluation of the host cytokine and chemokine response suggested activation of MDM by both wild-type Asibi virus and the 17D vaccine strain. Unlike what we previously observed in human Kupffer cells (resident liver macrophages) [16], wild-type YFV did not induce a significant and extended pro-inflammatory response, but rather both viruses induced a response that was broadly antiviral, with increases in IFNα and TNFα. Infection of human MDM with YFV also stimulated a chemotactic response evidenced through release of the chemokines MIP1α, MIP1β and RANTES. There were differences in the kinetics of release of the above cytokines and chemokines that largely reflected the virus propagation kinetics profiles for the respective viruses and may be associated with the infection efficiency of the two viruses or residual virus following washing of the cells. These data suggest that the cytokine and chemokine response is likely driven by increases in intracellular viral RNA potentially through recognition by pattern recognition receptors (PRR) such as RIG-I, MDA5 and TLR8. The processes by which these PRR function is well established and leads to activation of transcriptional regulators, such as NF-κB and IRF-7, that are critical for development of an innate immune response [25]. The limited pro-inflammatory response in MDM suggests that YFV infection may regulate or limit components of host innate immunity. The increase in chemokine release also suggests that YFV is able to stimulate recruitment of macrophages and T cells. However, our studies found that YFV infected MDM did not activate CD4^+^ T cells suggesting that YFV infection may limit the role of macrophages as APCs.

In NHP MDM the response to 17D virus infection was similar to what was seen in human MDM with evident increases in IFNα2 and TNFα, but little evidence of a pro-inflammatory response. The release of IFNα2 and TNFα did not mimic virus propagation kinetics as was seen in human MDM, but was a staged response with TNFα appearing early in the infection and IFNα2 increasing at 3–5 dpi. In Asibi virus-infected NHP MDM, there was little evidence of any cytokine response. Infection of NHP MDM with either Asibi or 17D viruses induced the release of MIP1α and MIP1β. In 17D virus-infected cells the MIP1α and MIP1β response was early in the infection (peaking with virus titers) and rapidly waned while in Asibi virus-infected cells the response was prolonged and didn’t diminish as the infection progressed. Interestingly, there was no evidence of RANTES secretion, which, in our experience, is unusual for virus infections. As was the case with human MDM, these data suggest activation of typical intracellular antiviral pathways, particularly those associated with a chemotactic response. The limited response of IFNα2 and TNFα in Asibi virus-infected cells suggests that this virus may inhibit some components of intracellular signaling, but given the limited response difference between Asibi and 17D virus-infected cells, it is not clear that any inhibitory response is significant.

The cytokine response to YFV infection in both human and NHP MoDCs was largely muted, with very few differences between virus- and mock-infected cells. In NHP MoDCs, the only significant difference was an apparent inhibition of the constitutive expression of IL-12p40 in Asibi virus-infected cells. While this result could be indicative of an impact on T cell differentiation, in the absence of other biological data, it is difficult to interpret.

In human MoDC, IL-1β was elevated in cells infected with either the 17D vaccine virus or the wild-type Asibi virus. The chemokines RANTES and IP-10 were elevated late following Asibi virus infection, but were not elevated in 17D virus-infected cells. The increase in IL-1β suggests activation of TLR signaling through endosomal TLR8 binding to viral ssRNA and subsequent activation of NF-κB which would drive expression of RANTES and IP-10 [26]. The IL-1 precursor is induced following activation of TLR/RIG-I like receptors and NF-κB, but cleavage of the precursor into its active form requires cleavage by caspase1 [27] that is a part of the inflammasome activated by PRR activation. Caspase-1 is also associated with activation of apoptosis; however, there was no significant evidence of cell death in any of the YFV-infected MoDC.

Fernandez-Garcia et al also examined the cytokine response in their studies with immortalized cells and immature MoDC [24]. Given that the cells tested in the Fernandez-Garcia study have biologically different roles during an infection than those used in this study, and none of the cytokine analytes were the same, it is difficult to make direct comparisons of the induced immune response between the two studies.

Through the use of re-stimulation assays, we were able to test the ability of YFV-infected APC to interact with and stimulate CD4^+^ T cells. We found that YFV-infected MDM did not stimulate CD4^+^ T cells, a result that is not surprising given that macrophages in general are considered less efficient as APCs than are dendritic cells. YFV-infected MoDCs, however, were able to stimulate CD4^+^ T cells and there were critical differences in the measured IFN-γ and IL-2 responses in these cells. In our studies, primary infection of MoDC with live 17D virus resulted in significantly higher percentages of IFN-γ producing CD4^+^ T cells than stimulation with MoDC infected with live Asibi virus, but this response may be associated with virus infection efficiency or residual virus in the assay system. The elevated IFN-γ response in the context of YFV 17D infected MoDC was consistent regardless of the secondary stimulation. In these studies, it is also evident that viral replication, or persistent stimulation, is required for CD4^+^ T cell activation as there was no significant elevation in IFN-γ or IL-2 responses in cells stimulated with inactivated YFV. These results conflict with studies by Moser *et al* and Gaucher *et al* who found that UV inactivated 17D YFV induced IL-2 expression in re-stimulated CD4^+^ T cells [22, 28]. The principal difference between the studies is the method of virus inactivation. The different mechanisms of inactivation could have a significant impact on interaction between viral RNA and PRR, or other molecules, within the cell. UV inactivation cross-links RNA to itself or closely associated proteins while the use of ionizing radiation induces nicks or breaks in the viral RNA. Both methods will block virus replication, but processing of the endocytosed particle and activation of intracellular signaling pathways could be very different.

Three of the human donors evaluated during these studies had previously been vaccinated with the 17D vaccine, although all of the vaccinations occurred more than 3 years prior to study onset. In order to determine if there was an evident T cell memory response in these donors, we compared intracellular IFN-γ and IL-2 responses following re-stimulation between donors who had and had not been vaccinated for YFV. These data indicate that there was not a significant response difference between vaccinated and unvaccinated donors, despite there being significant differences between YFV-infected MoDC and mock treated cells. There was also no difference in response between cells re-stimulated with 17D virus or Asibi virus. While an evident memory response could be anticipated, the number of peripheral memory CD4^+^ T cells is small and their response could have been insufficient to be measured in the context of these studies without additional enrichment processes.

The ability of virus infection to inhibit antigen presentation or activation of T cells is not unprecedented as a number of viruses have devised means to inhibit antigen presentation or MHC expression [29–33]. The studies completed here suggest that wild-type Asibi YFV may have a means of inhibiting the interaction between MoDC and CD4^+^ T cells. There are a number of amino acid differences between the 17D and the Asibi viruses [23], but the functional differences induced by these mutations are unknown. Structural analysis of the virus receptor-binding domain identified several amino acid differences between YFV 17D and Asibi that could directly affect receptor interaction, efficiency of infection and, subsequently, the ability of infected MoDC to stimulate T cells. In addition, if wild-type YFV is capable of inhibiting antigen presentation in the context of MHC class II and a mutation within the genome of the vaccine virus negates this capability, this could explain, in part, why the 17D virus is such an effective vaccine and why co-morbidities affecting T cells may be related to development of vaccine related disease. There are also some differences in the cytokine response in infected MoDCs, but none of these markers are obvious potential regulators of T cell responsiveness. It is possible that in the context of the co-culture environment, there are additional interactions among MoDC and CD4^+^ T cells that are not apparent in monoculture systems.

In the studies presented here, research efforts focused on potential differences in the interaction of APCs with wild-type or vaccine YFV. In these studies we developed a process to assure nearly homogeneous populations of APCs and demonstrated that wild-type and vaccine YFV could infect and replicate within these cells. We also found that YFV replication kinetics in human- or NHP-derived cells were remarkably similar and that 17D vaccine virus appeared to infect APCs more efficiently than did the wild-type Asibi virus. Cytokine response differences in infected APCs were marginal, but there were clear differences in the ability of infected MoDC to stimulate CD4^+^ T cells. These differences could be critical toward our understanding of YFV pathogenesis, the effectiveness of the 17D vaccine and potential high-risk co-morbidities that would preclude an individual from being vaccinated with the 17D virus. Clearly, there is significant work to be done toward determining if, in fact, antigen presentation is inhibited by wild-type YFV and, if so, the mechanisms associated with this discovery.

## Materials and Methods

### Viruses and cells

Wild-type YFV strain Asibi and the 17D-204 vaccine strain were obtained from Dr. Robert Tesh at the World Reference Collection for Emerging Viruses and Arboviruses (WRCEVA) at the University of Texas Medical Branch. Viruses were cultivated on Vero E6 cells (ATCC CRL-1586) in DMEM (Invitrogen) containing 10% heat-inactivated FBS (Sigma). Stocks were generated by infecting VeroE6 cells at a multiplicity of infection (MOI) of 0.1 (YFV 17D) or 0.01 (YFV Asibi) and allowing the cells to incubate at 37°C/5% CO_2_ until onset of cytopathic effects (4–7 days). The cell culture supernatants were collected and clarified by centrifugation at 500xg prior to aliquoting and storage at -80°C. Virus stocks used for these studies were passage two from receipt from the WRCEVA.

HuH-7 cells were provided by Hideki Ebihara at the NIAID Rocky Mountain Laboratories) and were cultivated in RPMI-1640 supplemented with 10% FBS.

Conditioned medium for macrophage differentiation was generated from cultures of KPB-M15 cells (Dr. Atsunoba Hiraoka, International Patent Organism Depositary, National Institute of Technology and Evaluation, Japan) grown in RPMI-1640 supplemented with 10% FBS.

### Virus inactivation

Yellow fever virus was inactivated by gamma irradiation (5 Mrad) in a JL Shepherd 484R-2 ^60^Co source. Complete inactivation was verified by titration of inactivated virus stocks. Inactivated YFV was used in T cell co-culture studies (see below).

### Kinetics assays

Cells were plated at a density of 2x10^5^ cells per well in 48-well plates and infected at a MOI of 0.1 for 1h at 37°C. The inoculum was removed, cells were washed once with PBS and the media was replaced with RPMI (Lonza) containing 10% FBS. Cell culture supernatants were collected at the indicated time-points and frozen at -80°C until use.

### Virus titration

Viruses were titrated on HuH-7 cells by 10-fold serial dilution of virus stocks in RPMI-1640 with 2% FBS. One-hundred μl of diluted virus was added to cells in a six-well plate and allowed to incubate for 1h at 37°C/5% CO_2_ with routine rocking. A semi-solid overlay, 0.8% gum tragacanth (f/c) in 1x EMEM containing 2% FBS, was added to the cells and the plates incubated undisturbed for 5 days at 37°C/5% CO_2_. The overlay was then removed and the cells fixed with neutral buffered formalin containing 0.25% crystal violet for 1h at room temperature. The fixed cell monolayers were washed with water and the plaques enumerated.

### PBMC isolation and purification

Human whole blood from healthy donors was obtained as a commercial resource through an agreement with the National Cancer Institute (NCI) in Frederick, MD and allowed repeated acquisition of blood from consistent donors. This attribute of NCI’s donor program allowed testing on material from the same donors throughout the study. Whole blood from NHPs was obtained from animals held within NIAID and also allowed for repeated acquisition of blood from the same individuals.

PBMCs were isolated from both human and NHP whole blood using Histopaque density gradient purification. Briefly, blood was diluted in PBS (pH 7.2) and layered over Histopaque (density 1.077 g/ml) (Sigma Chemical) and centrifuged at room temperature (RT) at 1000 xg for 10 minutes with the brake off. The interface was collected, diluted to 50 ml with PBS and then centrifuged at RT for 10 minutes at 300 xg with the brake on. The pellet was re-suspended in 50 ml PBS containing 2% FBS (v/v) prior to pelleting at RT for 10 min at 300 xg. The PBS/FBS wash was repeated twice more. Following the final wash, cells were counted and plated as required. In some instances, particularly with NHP PBMCs due to limited availability, cells were frozen using Recovery Cell Culture Freezing medium (Life Technologies) and stored in liquid nitrogen before use.

### Macrophage and dendritic cell differentiation

CD14^+^ cells were isolated from bulk PBMCs using species-specific magnetic microbead enrichment kits (Miltenyi Biotec) following the manufacturer’s instructions.

To differentiate CD14^+^ monocytes into dendritic cells, monocytes were re-suspended in RPMI-10 containing 20 ng/ml GM-CSF and 10 ng/ml IL-4 and incubated for 6 days at 37°C/5% CO_2_ with a change of media every second day. On day 6 post-plating, the media was changed to DC maturation cocktail (RPMI-10 containing 10 ng/ml TNF-α, 10 ng/ml IL-1β, 15 ng/ml IL-6, 1 ug/ml prostaglandin E2, 20 ng/ml GM-CSF and 10 ng/ml IL-4) and incubated overnight at 37°C/5% CO_2_. Non-adherent cells were washed with cold PBS and collected. Adherent cells were collected using enzyme-free Dissociation Buffer (Gibco) and incubated for 5–15 min at 37°C/5% CO_2_. Cells were pelleted by centrifugation at 300 xg for 10 min at RT, re-suspended and combined prior to counting, plating and characterization by flow cytometry.

To differentiate CD14^+^ monocytes into macrophages, monocytes were re-suspended in macrophage culture medium (50% RPMI-10, 50% KPB-M15 conditioned medium and 10 ng/ml M-CSF). Cells were cultured at 37°C/5% CO_2_ for 7 days with feeding on days 2, 4 and 6 by removal and replacement of the medium. Cells were harvested in cold PBS containing 2 mM EDTA.

### APC phenotyping and analyses

MDM and MoDC were characterized by flow cytometry. Briefly, the harvested cells were washed once with PBS containing 2% FBS. The cells were then incubated at room temperature with fluorochrome-conjugated monoclonal antibodies diluted in PBS/ 2% FBS for 20 minutes while protected from light. The cells were washed again, and the resulting cell pellet re-suspended in PBS/ 2% FBS. The sample data were acquired using BD LSR II Fortessa flow cytometer equipped with BD FACSDiva software. The antibody panel for MDM included: anti-CD11b (ICRF44)-Pacific Blue, anti-CD11c (N418)-AF700, anti-CD14 (M5E2)-FITC, anti-CD86 (2331 (FUN-1))-PE-Cy7, anti-CD163 (GHI61)-APC, anti-CD206 (15–2)-PE-Cy5, anti-HLA-DR (G46-4)-V500 and yellow VID live-dead stain. The MoDC panel included: anti-CD14 (M5E2)-PE, anti-CD40 (5C3)-FITC, anti-CD80 (L307.4)-Qdot605, anti-CD83 (HB15e)-PerCP-Cy5.5, anti-HLA-DR (G46-4)-V500 and yellow VID live-dead stain. Antibodies were purchased from either BD Biosciences or BioLegend. Yellow viability dye was purchased from Invitrogen. Data was analyzed using Community Cytobank data analysis software (Cytobank).

### Analysis of cytokines from infected MDM and MoDC

Cell culture supernatants collected during kinetics assays were tested for secretion of a panel of cytokines using custom designed 16-plex magnetic bead multi-analyte panels (Millipore). Milliplex custom cytokine kits included a 16-plex human panel (GM-CSF, TNF-α, IL-1RA, IL-1β, IL-5, IL-6, IL-10, IL-12/23(P40), IL-13, IL-15, IL-17A, MIP-1α, MIP-1β, IFNα2, IP-10, and RANTES) and a 14-plex NHP panel (GM-CSF, TNF- α, IL-1RA, IL-1β, IL-5, IL-6, IL-10, IL-12/23(P40), IL-13, IL-15, IL-17A, IL-18, MIP-1 α, MIP-1β) supplemented with a 3-plex human panel (IFNα2, IP-10, and RANTES) that is cross-reactive with rhesus macaque antigens. TGF-β assays were not available in multi-plex format; single bead kits were purchased from Millipore. All reagents were prepared according to the manufacturers' instructions and assays completed in 96-well mylar plates (Millipore). Fluorescent-coded magnetic beads microspheres coated with a capture antibody were incubated with cell culture supernatant test material. The volume of concentrated beads required for each assay was determined using the protocol chart provided by the manufacturer and diluted to the correct volume using bead diluent. The plates were assayed on a Luminex FLEXMAP 3D system equipped with xPonent 4.2 software (Luminex Corp). The data collection software was set to acquire data by collecting an average of 50 beads per analyte per well in a volume of 100 μl. The raw data was measured as mean fluorescence intensity (MFI) and the concentration of each analyte was calculated using a 4- or 5-parameter logistic fit curve generated for each analyte from the 6 standards. The lower limit of quantification (LLOQ) was determined using the lowest standard that was at least 3 times above background. The calculation of the LLOQ was performed by subtracting the MFI of the background (diluent) from the MFI of the lowest standard concentration and back-calculating the concentration from the standard curve. Data were further analyzed using Prism 6.0 software (GraphPad).

### T cell co-culture assay

Human MoDCs or MDM were isolated as described above and treated with each of the following stimulants: live Asibi virus, inactivated Asibi virus, live 17D virus, inactivated 17D virus, or mock stimulated with media diluent. Following addition of the stimulant, cells were incubated for 1h at 37°C. Cells were then washed with PBS to remove residual virus. Autologous CD4^+^ T cells were isolated using CD4 specific microbeads (Miltenyi Biotec) to positively select CD4^+^ populations. The CD4^+^ T cells were then added to the APC in a ratio of 60:1 (T cell:APC) and allowed to incubate for 14d at 37°C/5% CO_2_. At the end of the primary stimulation, the T cells were purified from the co-culture and mixed with fresh autologous APCs that had been stimulated as indicated above. The secondary stimulation was continued with incubation of the co-culture at 37°C/5% CO_2_ for 7h, with 1ug/ ml Brefeldin A (Sigma) added for the final 5h of the incubation. Cells were then collected and stained with live/ dead cell stain kit (Invitrogen) along with surface staining for CD3, CD4 for 20 min at room temperature in the dark. The cells were washed, fixed and permeabilized using cytofix/cytoperm (BD Biosciences), with intracellular staining performed by incubating permeabilized cells with anti-IFN-γ and anti-IL-2 specific fluorophore-conjugated antibodies at RT for 1 hour [22, 28]. The cells were then washed twice prior to acquisition.

The antibody panel used for re-stimulated T cells included: anti-CD3 (SP34-2)-v500, anti-CD4 (S3.5)-APC-AF750, anti-IL-2 (MQ1-17H12)-APC, anti-IFNγ (B27)-PE-Cy7 and yellow VID live-dead stain. Antibodies were purchased from either BD Biosciences, Invitrogen, eBiosciences or BioLegend. Yellow viability dye was purchased from Invitrogen. Data was analyzed using Community Cytobank data analysis software (Cytobank). The gating strategy used for analyzing re-stimulated T cells is included in S6 Fig.

### Statistics

GraphPad Prism 6.0 was used for determination of statistical significance and graphical representations. The non-parametric multi-T test built into GraphPad Prism 6.0 was applied to determine the significance of T cell responses in APC co-culture studies. Repeated measures ANOVA run in SAS was used to determine statistical significance in multiplex cytokine analyses in human/NHP MDM and MoDC infected with YFV. In all analyses, p values < 0.05 were considered significant.

### Ethics statement

Acquisition and use of human material utilized in these studies was exempt from NIAID IRB approval, but met all requisite approvals at the NCI, Frederick, a commercial resource. The acquisition of whole blood from non-human primates was conducted in accordance with an Animal Study Protocol approved by the NIAID Division of Clinical Research Animal Care and Use Committee (Protocol #IRF-001) following recommendations in the Guide for the Care and Use of Laboratory Animals of the National Institutes of Health. This institution also accepts as mandatory the PHS policy on Humane Care of Vertebrate Animals used in testing, research and training. All animal work at NIAID is performed in a facilities accredited by the American Association for the Accreditation of laboratory Animal Care.

Non-human primates were housed either singly or in pairs in an ABSL-2 facility with appropriate enrichment including, but not limited to, polished steel mirrors and durable toys. Animals were anesthetized prior to collection of blood to minimize stress to the animals. Animals were observed following blood collection to ensure recovery from the anesthesia. All work with non-human primates was done in accordance with the recommendations of the Weatherall Report.

## Supporting Information

S1 Fig YFV 17D kinetics on human PBMCs and NHP imDCs. YFV 17D propagation kinetics were measured in human derived bulk PBMCs (■) or immature NHP DCs (●), each from an individual donor. Titrations were performed in triplicate with data points representing the mean of the triplicate values.
(TIF)

S2 Fig Measurement of YFV titers before and after wash. Virus titers from MoDC and MDM from three human donors were measured one hour after addition of YFV inoculum and immediately after one wash with PBS. Each data point represents an individual donor. The assay was performed with triplicate samples.
(TIF)

S3 Fig Molecular model of YFV Envelope protein domain III from YFV 17D and Asibi. Molecular models developed by the Swiss-Model server based on submitted amino acid sequences for YFV 17D and Asibi. The top two panels highlight mutations at the top of domain III which would be the exposed virus surface. The bottom two panels provide a top-down view of the same amino acid changes. The amino acids present at the specific residues are indicated.
(TIF)

S4 Fig Cytokine response in CD4^+^ T cells: Wild-type Asibi virus vs. vaccine 17D virus infection-co-cultured with MDM. IFN-γ and IL-2 production by human CD4^+^ T cells in re-stimulation assays. Each data point represents the response from an individual donor (n = 6) with the horizontal bar indicating the mean of the six values. Red data points indicate 17D YFV-treated cells, green squares indicate Asibi YFV-treated cells and yellow triangles indicate mock-treated cells. (L) indicates treatment with live virus, (D) indicates treatment with gamma-irradiated inactivated virus and (N) indicates mock-treated MDM prior to co-culturing with CD4^+^ T cells (See Fig 7 and Materials and Methods). (*) indicates points of significant (p<0.05) difference between the indicated datasets (bracket). A non-parametric multi-T test was used to determine statistical significance.
(TIF)

S5 Fig Cytokine response in CD4^+^ T cells: Vaccinated vs. unvaccinated-co-cultured with MDM. IFN-γ and IL-2 production by human CD4^+^ T cells in re-stimulation assays. Each data point represents the response from an individual donor (n = 6) with the horizontal bar indicating the mean of the six values. Red circles indicate cells isolated from vaccinated donors and green squares indicate cells isolated from unvaccinated donors. Yellow triangles indicate mock-treated (N+N) control cells. (L) indicates treatment with live virus, (D) indicates treatment with gamma-irradiated inactivated virus and (N) indicates mock-treated MDM prior to co-culturing with CD4^+^ T cells (See Fig 7 and Materials and Methods). (*) indicates points of significant (p<0.05) difference between the indicated datasets (bracket). A non-parametric multi-T test was used to determine statistical significance.
(TIF)

S6 Fig Gating strategy for analysis of re-stimulated CD4^+^ T cells. All cells in culture were collected and gated specifically on viable singlet CD3^+^ CD4^+^ T cell populations. Analysis of IFN-γ and IL-2 expression was completed only on CD4^+^ T cells. The data presented are from a representative sample.
(TIF)
