# Supplementary figures and images for: Characterization of Yellow Fever Virus Infection of Human and Non-human Primate Antigen Presenting Cells and Their Interaction with CD4+ T Cells
Source: PLoS Negl Trop Dis. 2016 May 18;10(5):e0004709. doi: 10.1371/journal.pntd.0004709 (PMC4871483; doi:10.1371/journal.pntd.0004709)

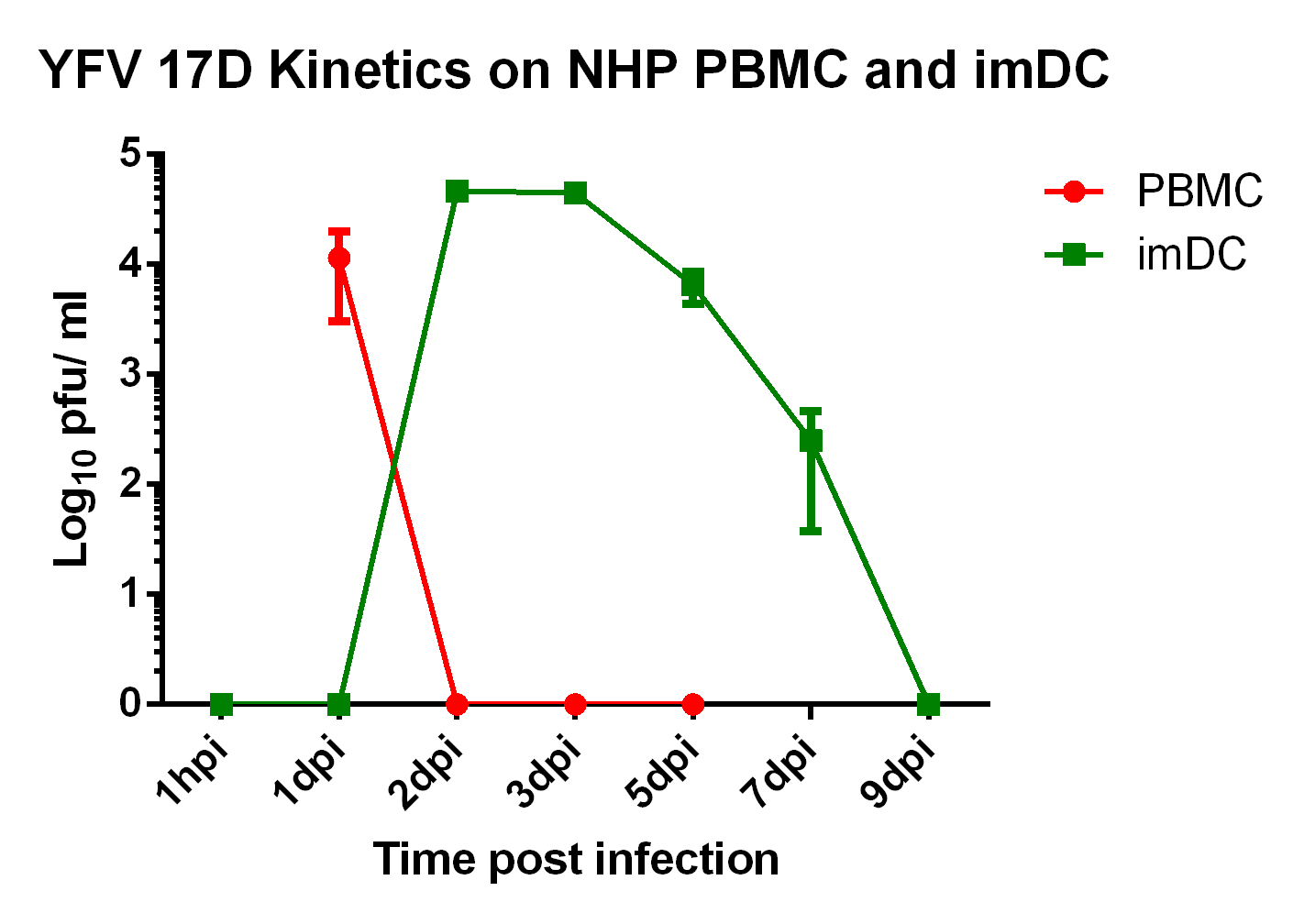

Supplement: S1 Fig — YFV 17D propagation kinetics were measured in human derived bulk PBMCs (■) or immature NHP DCs (●), each from an individual donor. Titrations were performed in triplicate with data points representing the mean of the triplicate values. (TIF) [file pntd.0004709.s001.tif]

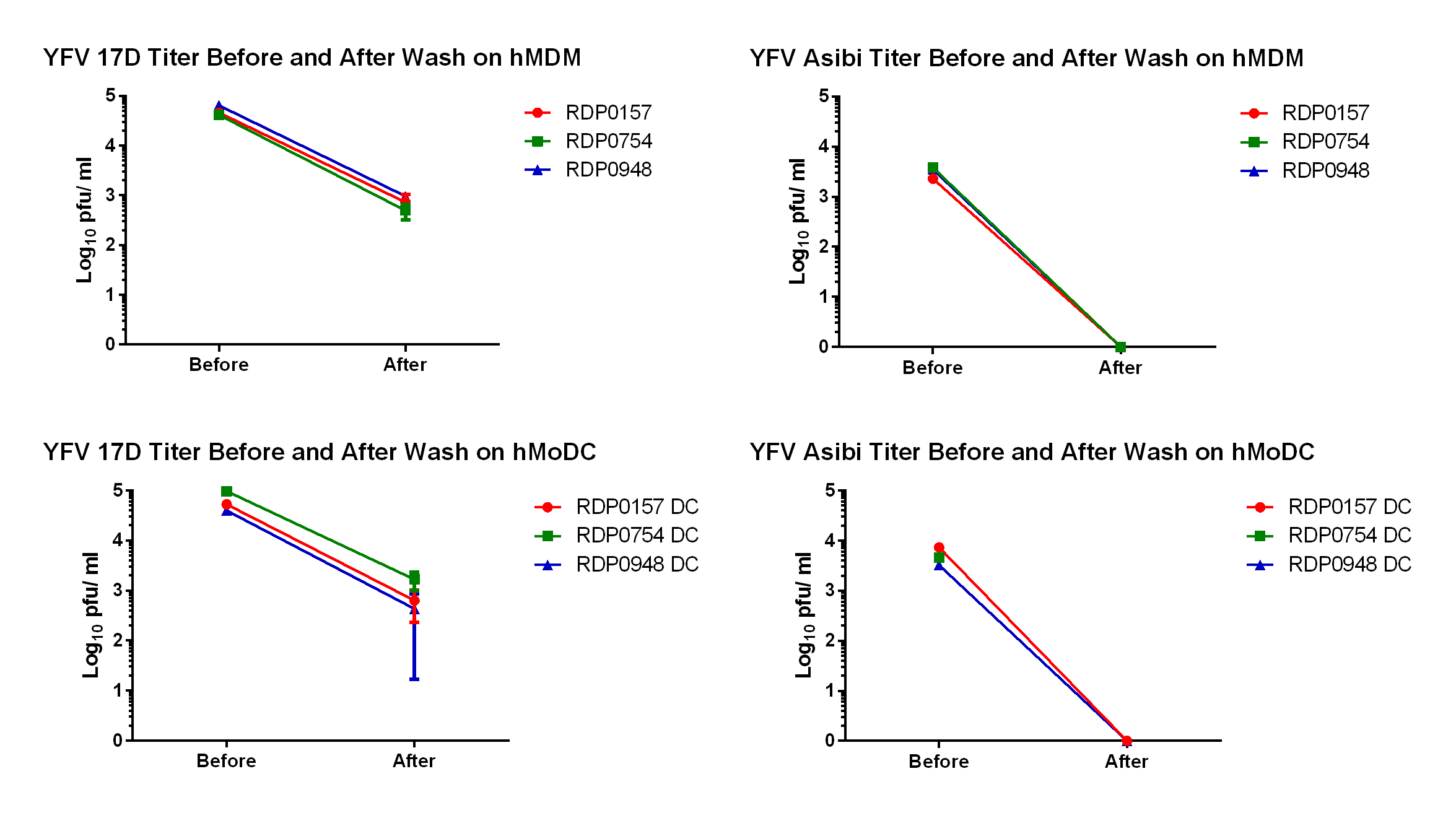

Supplement: S2 Fig — Virus titers from MoDC and MDM from three human donors were measured one hour after addition of YFV inoculum and immediately after one wash with PBS. Each data point represents an individual donor. The assay was performed with triplicate samples. (TIF) [file pntd.0004709.s002.tif]

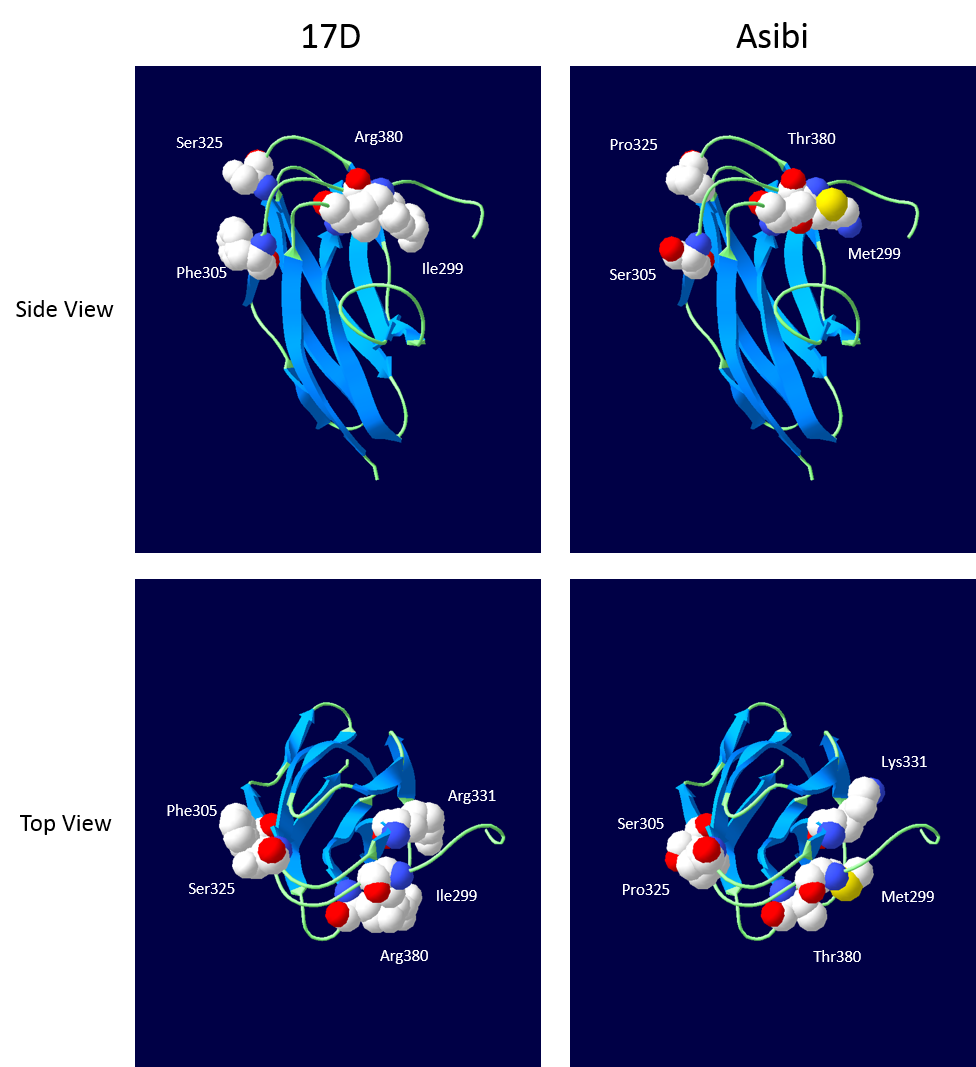

Supplement: S3 Fig — Molecular models developed by the Swiss-Model server based on submitted amino acid sequences for YFV 17D and Asibi. The top two panels highlight mutations at the top of domain III which would be the exposed virus surface. The bottom two panels provide a top-down view of the same amino acid changes. The amino acids present at the specific residues are indicated. (TIF) [file pntd.0004709.s003.tif]

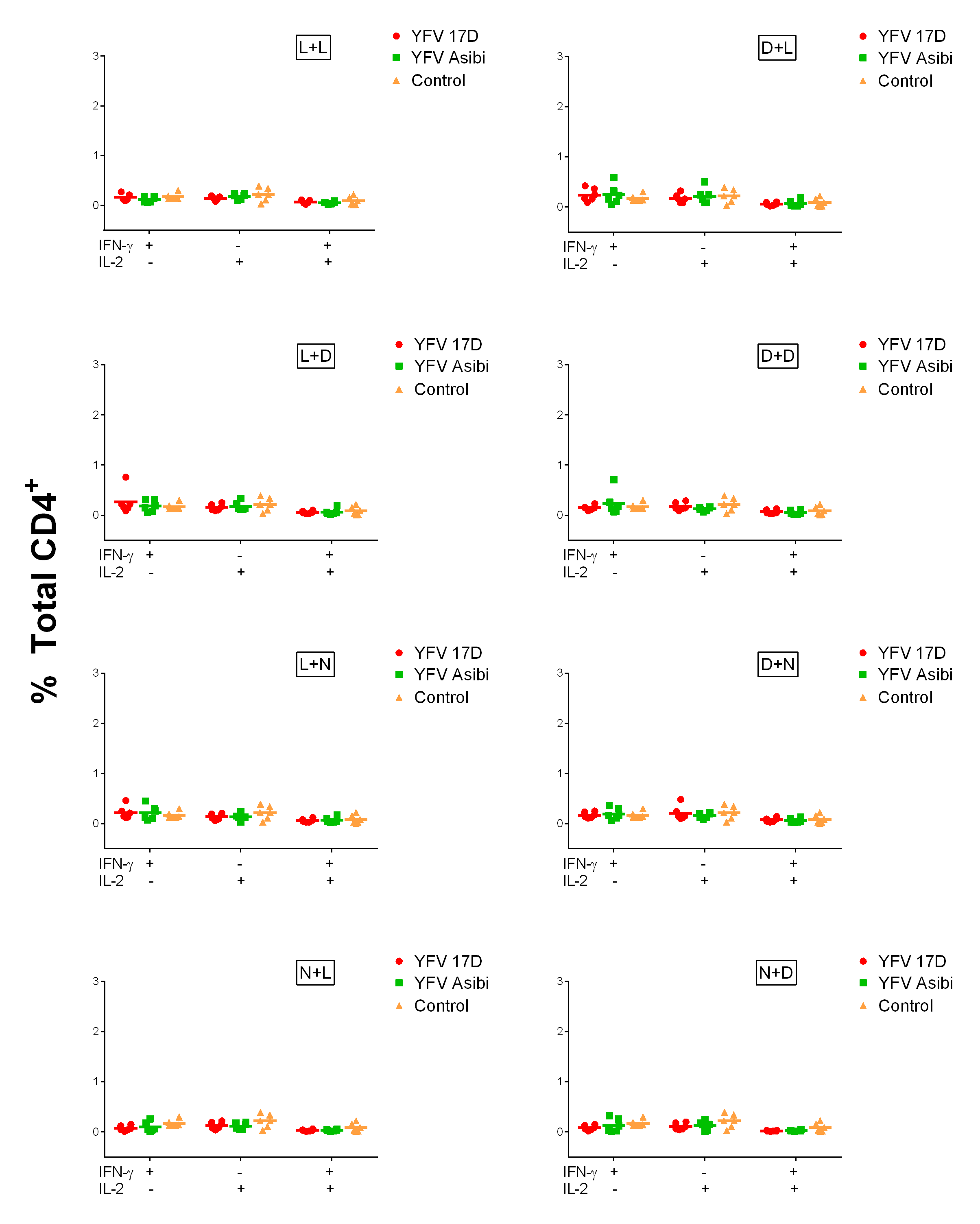

Supplement: S4 Fig — IFN-γ and IL-2 production by human CD4+ T cells in re-stimulation assays. Each data point represents the response from an individual donor (n = 6) with the horizontal bar indicating the mean of the six values. Red data points indicate 17D YFV-treated cells, green squares indicate Asibi YFV-treated cells and yellow triangles indicate mock-treated cells. (L) indicates treatment with live virus, (D) indicates treatment with gamma-irradiated inactivated virus and (N) indicates mock-treated MDM prior to co-culturing with CD4+ T cells (See Fig 7 and Materials and Methods). (*) indicates points of significant (p<0.05) difference between the indicated datasets (bracket). A non-parametric multi-T test was used to determine statistical significance. (TIF) [file pntd.0004709.s004.tif]

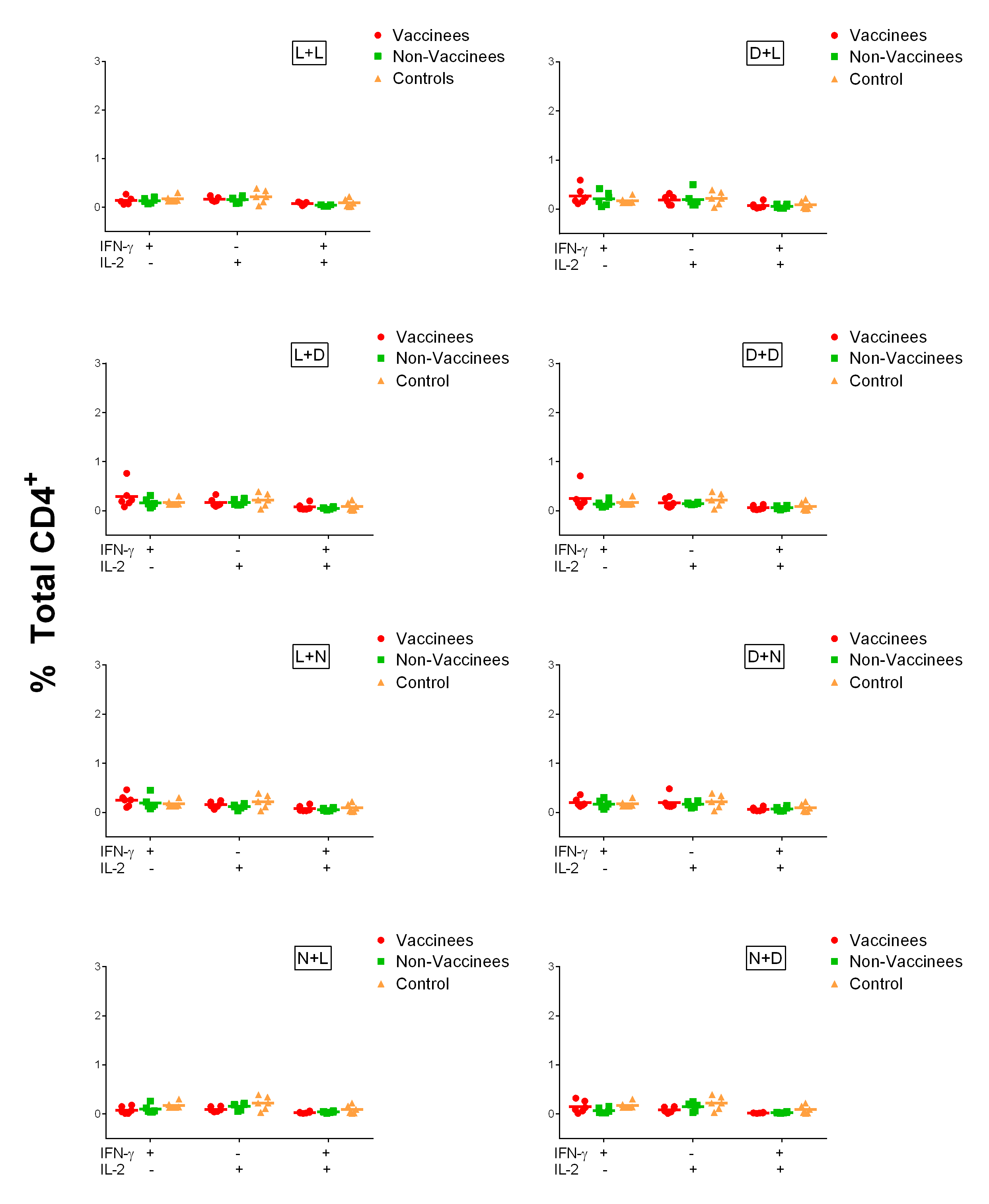

Supplement: S5 Fig — IFN-γ and IL-2 production by human CD4+ T cells in re-stimulation assays. Each data point represents the response from an individual donor (n = 6) with the horizontal bar indicating the mean of the six values. Red circles indicate cells isolated from vaccinated donors and green squares indicate cells isolated from unvaccinated donors. Yellow triangles indicate mock-treated (N+N) control cells. (L) indicates treatment with live virus, (D) indicates treatment with gamma-irradiated inactivated virus and (N) indicates mock-treated MDM prior to co-culturing with CD4+ T cells (See Fig 7 and Materials and Methods). (*) indicates points of significant (p<0.05) difference between the indicated datasets (bracket). A non-parametric multi-T test was used to determine statistical significance. (TIF) [file pntd.0004709.s005.tif]

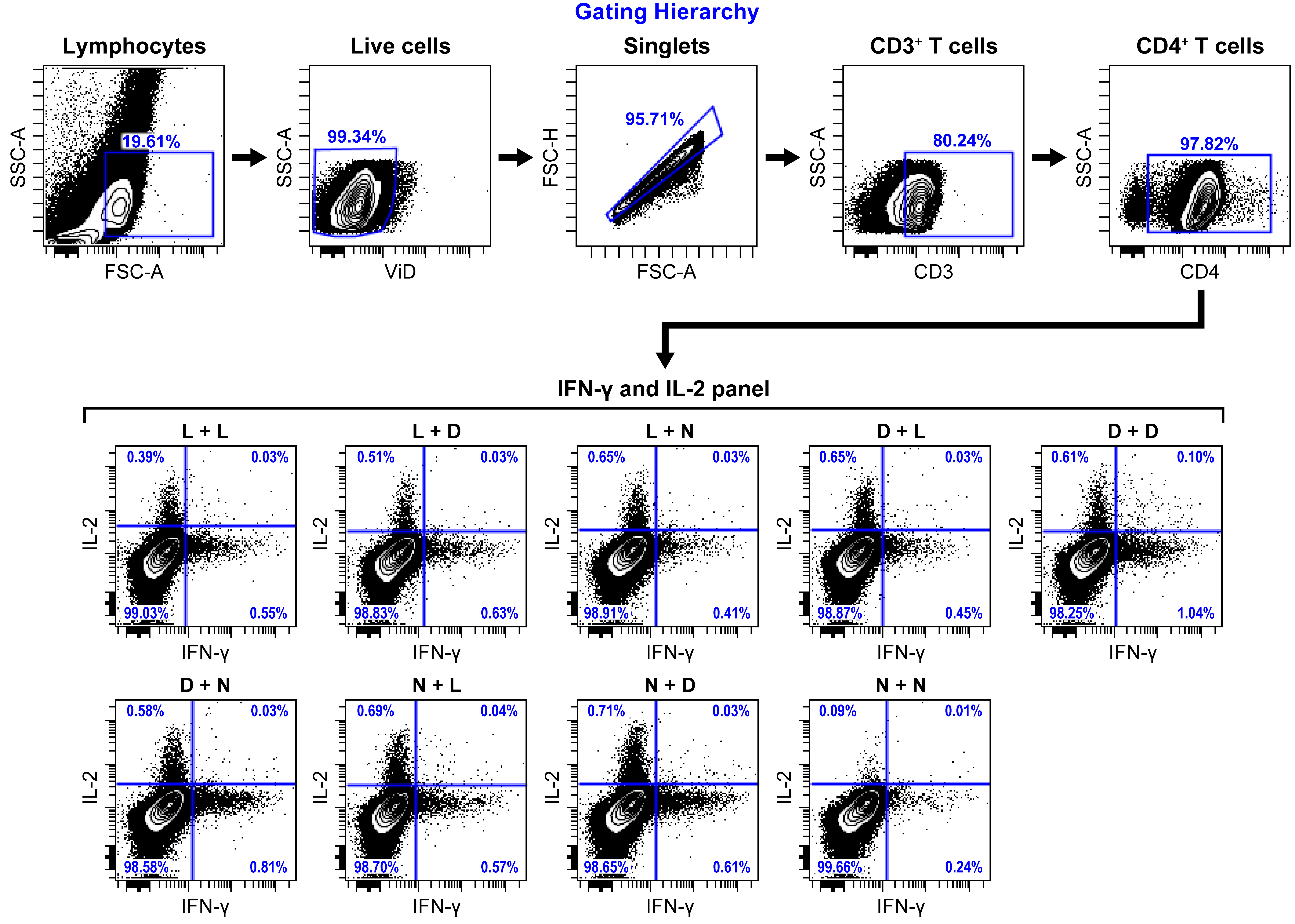

Supplement: S6 Fig — All cells in culture were collected and gated specifically on viable singlet CD3+ CD4+ T cell populations. Analysis of IFN-γ and IL-2 expression was completed only on CD4+ T cells. The data presented are from a representative sample. (TIF) [file pntd.0004709.s006.tif]
